# Improvement of genome editing efficiency by Cas9 codon optimization in Japanese cedar (*Cryptomeria japonica* D. Don)

**DOI:** 10.5511/plantbiotechnology.24.0709a

**Published:** 2024-12-25

**Authors:** Yoshihiko Nanasato, Harunori Kawabe, Saneyoshi Ueno, Ken-ichi Konagaya, Masaki Endo, Toru Taniguchi

**Affiliations:** 1 Forest Bio-Research Center, Forestry and Forest Products Research Institute (FFPRI), Forest Research and Management Organization (FRMO), 3809-1 Ishi, Juo, Hitachi, Ibaraki 319-1301, Japan; 2 Department of Forest Molecular Genetics and Biotechnology, FFPRI, FRMO, 1 Matsunosato, Tsukuba, Ibaraki 305-8687, Japan; 3 Institute of Agrobiological Sciences, National Agriculture and Food Research Organization, 1-2 Owashi, Tsukuba, Ibaraki 305-8634, Japan

**Keywords:** codon optimization, CRISPR/Cas9, *Cryptomeria japonica*, genome editing, Japanese cedar

## Abstract

Japanese cedar or sugi (*Cryptomeria japonica* D. Don) is among the most important plantation conifers in Japan, occupying 12% of the total land area in the country. We have successfully established a CRISPR/Cas9-based genome editing system in *C. japonica*. However, in practical use, we encountered problems of low efficiency when generating biallelic mutations, i.e., target gene knockouts. As part of our efforts to improve efficiency, we codon-optimized the Cas9 gene, evaluated by the genome editing efficiency of *CjChl I*, a gene encoding a chlorophyll biosynthesis enzyme. As a result, our codon-optimized SpCas9, named ^Cj^SpCas9, performed the highest genome editing efficiency of two targets (t4, t1+t2). Specifically, the biallelic disruption efficiency of the *CjChl I* with ^Cj^SpCas9 was 1.8-fold higher than that of the SpCas9 gene optimized for *Arabidopsis thaliana* (^At^SpCas9) and 2.0-fold higher than that of the SpCas9 gene optimized for *Orysa sativa* (^Os^SpCas9) for t4, respectively. For t1+t2, the efficiency was 4.9-fold higher than that of ^At^SpCas9 and 1.4-fold higher than that of ^Os^SpCas9, respectively. Our western blotting analysis proved that the Cas9 protein accumulation increased upon codon frequency optimization. We concluded that the observed efficiency improvement was due to the increased Cas9 protein quantity. The efficient genome editing system we report here would accelerate molecular breeding in conifers.

## Introduction

The Japanese cedar or sugi (*Cryptomeria japonica* D. Don) is a conifer species of the Cupressaceae family, distributed across Japan. It is the dominant tree species in Japanese planted forests, covering 12% of the land in Japan (Forestry Agency Japan 2019). The national breeding program on economically important tree species, including *C. japonica*, has been ongoing since the 1950s for selecting plus-trees, i.e., individuals with a better growth performance in forests, leading to the selection of more than 3,500 *C. japonica* plus-tree clones (Kondo and Kuramoto 2007). Now, the breeding program has been extended to improvement of wood properties, enhancement of CO_2_ storage capacity, and tolerance to meteorological damage (Takahashi et al. 2023). Meanwhile, due to the relevant quantity of *C. japonica* pollen dispersed during spring, an estimated 38.8% of Japanese citizens suffer from allergic symptoms (Matsubara et al. 2020). During *C. japonica* pollinosis, representing a severe public health concern, non-pollen or male sterility is an important breeding target along with forestry traits (e.g., growth and wood properties). The discovery of a male-sterile *C. japonica* tree in Toyama Prefecture in 1992 (Taira et al. 1993) paved the way for breeding male-sterile plus-tree lines. Previous studies described that *C. japonica* male sterility is controlled by several recessive genes (i.e., *MS1–4*) from test crossing (Moriguchi et al. 2012, 2014, 2016). Based on linkage map information, these male-sterile genes have been mapped on linkage groups (LG) 9, 5, 1, and 4, respectively (Hasegawa et al. 2018). DNA markers for male-sterile genes have been developed to enable the rapid discrimination of male-sterile individuals (Hasegawa et al. 2020; Mishima et al. 2018). In addition, the causative genes of *MS1* and *MS4* have been identified (Hasegawa et al. 2021; Kakui et al. 2023).

Technical advances in next-generation sequencing allowed for deciphering large and complicated genome sequences of important coniferous trees in Japan (e.g., *C. japonica*, *Chamaecyparis obtusa*, *Larix kaempferi*, and *Cunninghamia lanceolata*) (Fujino et al. 2023; Shirasawa et al. 2023). Targeted mutagenesis- or genome editing-mediated molecular breeding of such coniferous trees is emerging upon the development of various genome editing tools, such as meganucleases, zinc-finger nucleases, transcription activator-like effector nucleases, and CRISPR/Cas9 (Cermak et al. 2011; Jinek et al. 2012; Osakabe et al. 2010; Puchta et al. 1996). Genome editing in forest trees was first reported in the genus *Populus* using the CRISPR/Cas9 system in 2015 (Fan et al. 2015; Zhou et al. 2015). Thereafter, genome editing in several coniferous trees, including *C. japonica*, *Picea glauca*, and *Pinus radiata*, was described successively in 2021 (Cui et al. 2021; Nanasato et al. 2021; Poovaiah et al. 2021).

The CRISPR/Cas9-mediated knockout of the endogenous magnesium chelatase subunit I (*CjChl I*) gene in *C. japonica*, involved in chlorophyll synthesis allowed for biallelic mutation-associated albino plant generation (Nanasato et al. 2021). However, knockout individual generation efficiency has been limited, leading to the acquisition of only one transgenic line with biallelic mutations in each CRISPR/Cas9 vector. We considered that insufficient Cas9 protein expression and accumulation was the reason for this limitation, which could be remedied by codon frequency optimization to align with the host organism (Bai et al. 2011; Kwon et al. 2016). Codon frequency optimization is reportedly effective in genome editing efficiency enhancement (Li et al. 2013; Sugano et al. 2017). However, no such attempt has been described in coniferous trees. In this study, we present the codon frequency optimization of the SpCas9 gene in *C. japonica*, resulting in significant Cas9 protein accumulation enhancement and biallelic mutation generation efficiency improvement.

## Materials and methods

### Plant materials

We used a *C. japonica* embryogenic tissue (ET) cell line, designated as #13-8-12 as well as the GFP-overexpressing ET line N4 (Nanasato et al. 2021). We maintained the #13-8-12 and N4 cell lines in the dark at 25°C on solid 1/2MD medium (Konagaya et al. 2020) and solid 1/2MD medium supplemented with 5 mg l^−1^ hygromycin, respectively. We subcultured both ET lines for 2 weeks on fresh media.

### SpCas9 codon optimization in *C. japonica*

We determined the codon usage frequency in *C. japonica* by analyzing the related EST information in the ForestGEN database (https://forestgen.ffpri.go.jp/jp/index.html (Accessed Oct 3, 2024)). We predicted the coding sequences (CDSs) from 12,045 nonredundant full-length cDNA (FLcDNA) sequences (Supplementary Table S1) out of a total of 22,539 FLcDNA sequences derived from male cones, female flowers, needles, and reproductive shoots (Futamura, unpublished set) using the FrameDP program (Gouzy et al. 2009). Subsequently, we counted and summarized in a table the number of codons corresponding to each amino acid (Table 1). Based on the codon table, we codon-optimized the SpCas9 gene, fused with a nuclear localization signal at the 3′ terminal, for *C. japonica* and synthesized it by Invitrogen (GeneArt, Thermo Fisher Scientific, Waltham, MA, USA). Hereafter, we refer to the codon-optimized Cas9 gene as ^Cj^SpCas9.

**Table 1:** Table 1. Codon frequency table of *Arabidopsis thaliana*, *Oryza sativa*, and *Cryptomeria japonica*.

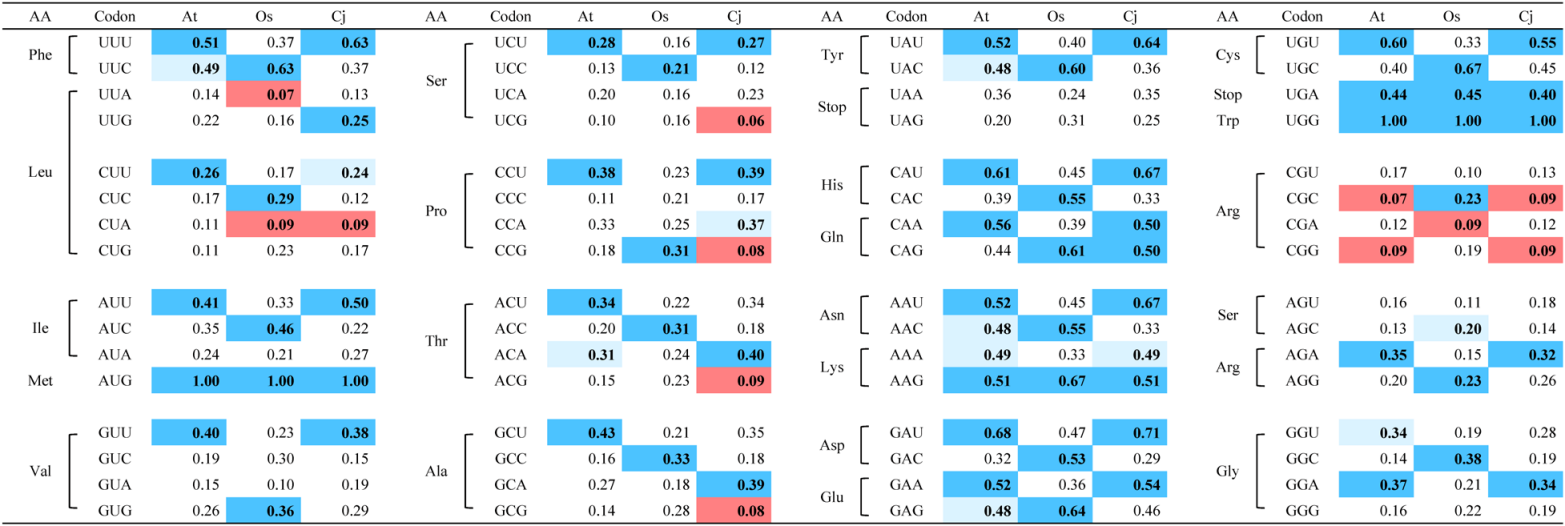

Codon usage tables were created with the information from Codon Usage Database (https://www.kazusa.or.jp/codon/ (Accessed Oct 3, 2024)) and Forest EST and Genome Database (http://forestgen.ffpri.affrc.go.jp/jp/index.html (Accessed Oct 3, 2024)). Codon with low frequency (<10%) are highlighten in red. The most preferred codon for each amino acid is highlighted in dark blue. The second most frequent codon for each amino acid is indicated in light blue, if it differs by less than 10% of the frequency of the first. Abbreviations: AA, amino acid; At, *Arabidopsis thaliana*; Os, *Orysa sativa*; Cj, *Cryptomeria japonica*.

### Vector construction

**Figure 1:**
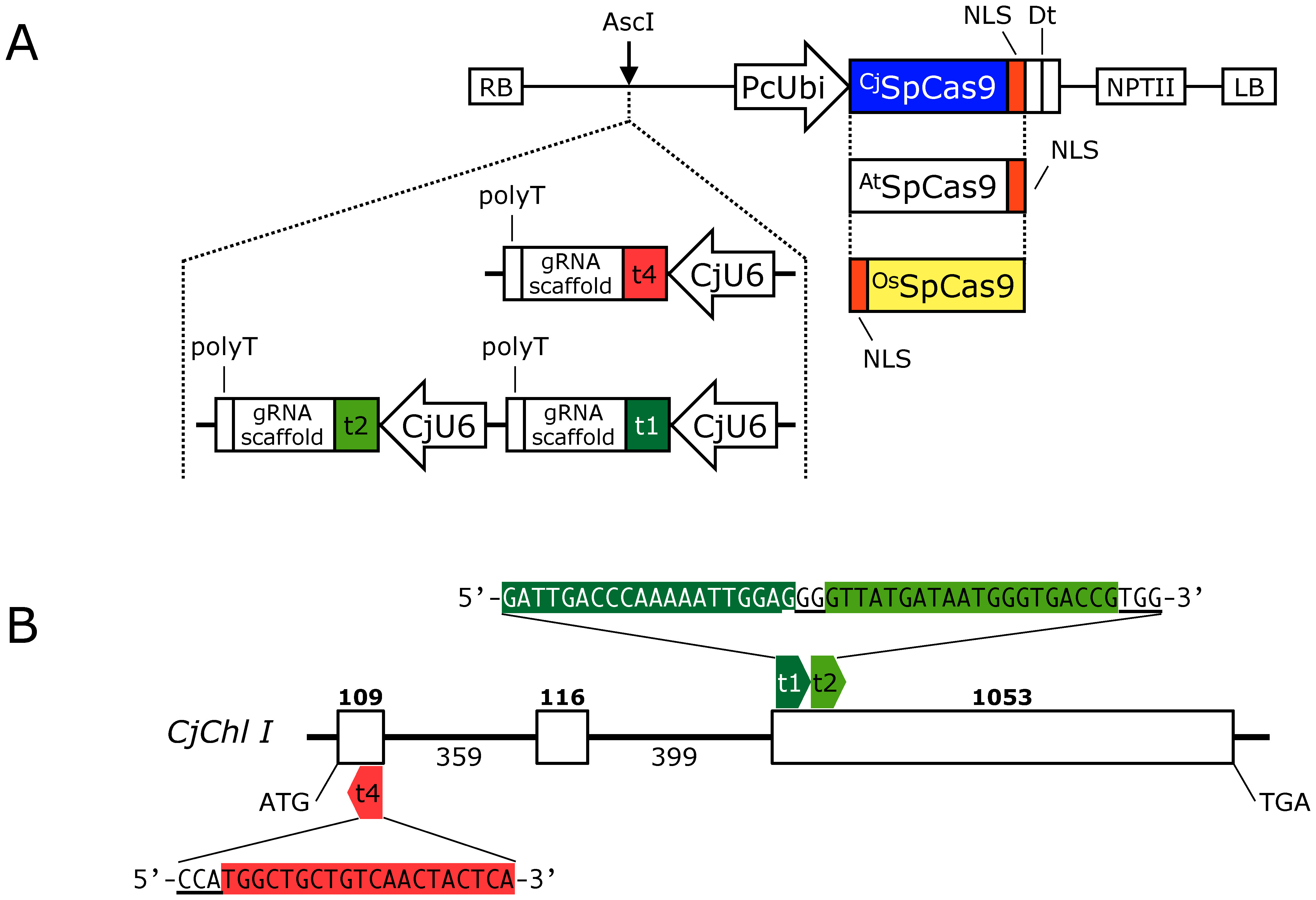
Figure 1. Gene editing of magnesium chelatase gene (*CjChl I*) using various SpCas9 genes. (A) Schematic diagram of pCRG-SpCas9-derived vectors. SpCas9 genes codon-optimized for *Cryptomeria japonica* (^Cj^SpCas9), *Arabidopsis thaliana* (^At^SpCas9), and *Oryza sativa* (^Os^SpCas9). The guide RNA-expression cassette was inserted into the AscI recognition site using the In-Fusion system. RB, right border; CjU6, *C. japonica* U6 polymerase III promoter #2; PcUbi, ubiquitin promoter from *Petroselinum crispum*; NLS, SV40 nuclear localization signal; Dt, tandem terminator unit of heat shock protein terminator from *Arabidopsis thaliana* and nopaline synthase terminator from *Agrobacterium tumefaciens*. (B) The structure of the *CjChl I* genome and target sequences. Bold and regular fonts indicate the number of exon and intron lengths, respectively. White boxes and lines represent exons and introns, respectively.

We constructed the CRISPR/Cas9 expression vectors (Figure 1) based on pCRG-SpCas9 (Nanasato et al. 2021). The SpCas9 gene in pCRG-SpCas9 originated from FFCas9, optimized for *Arabidopsis thaliana* in the pDe-CAS9 vector (Fauser et al. 2014), designated ^At^SpCas9, and we coined the vector pCRG-^At^SpCas9. We excluded the omega sequence from the original vector as it was reportedly ineffective in *C. japonica* (Nanasato et al. 2021). We obtained the codon-optimized *Oryza sativa* SpCas9 from the MMCas9 vector (Mikami et al. 2015) and named it pCRG-^Os^SpCas9. Moreover, we removed from the sequence the OsADH2 5′ UTR, which was initially fused to MMCas9, and replaced ^Cj^SpCas9 with ^At^SpCas9 in the pCRG-SpCas9 vector, resulting in the creation of pCRG-^Cj^SpCas9. We inserted the gRNA expression cassettes, driven by the *C. japonica* U6 polymerase III promoter #2 (CjU6_#2) for editing *GFP* or *CjChl I*, into the AscI site between the right border and the ubiquitin promoter from *Petroselinum crispum* (PcUbi) promoter (Kawalleck et al. 1993) in the vector using an overlapping PCR method as described previously (Nanasato et al. 2021). Briefly, we used the CjU6_#2-containing pCjU6_#2 as a template. For the GFP target #2_rev expression cassette, we performed a PCR using the primer pair p#474_f and p#333_r and another round of PCR with the primer pair p#475_r and p#343_f. We used KOD -Plus- Neo DNA polymerase (Toyobo, Osaka, Japan) for PCR amplification in a 20-µl PCR mixture. The PCR conditions were as follows: 94°C for 2 min; 30 cycles at 98°C for 10 s, 50°C for 30 s, and 68°C for 20 s; final extension at 68°C for 7 min. We purified the amplified fragments using the MinElute Gel Extraction Kit (QIAGEN, Venlo, Netherlands) and inserted them into AscI-cut pCRG vectors containing the respective SpCas9 using the In-Fusion HD cloning kit (Takara Bio Inc., Shiga, Japan). For the *CjChl I*_t4 expression cassette, we used two primer pairs (p#440_f and p#333; p#441_r and p#343) to amplify the two fragments. For tandem *CjChl I*_t1 and t2 expression cassette insertion, we amplified four fragments using the primer pairs as follows: #354_r and p#343_f; p#353_f and p#329_T3_r; p#355_f and p#333_r; p#356_r and p#347_T3_f. The PCR conditions and vector construction were identical to those above. Supplementary Table S2 summarizes the primers used in this study.

### *C. japonica* transformation

We produced the CRISPR/Cas9 expression cassette-containing ET cell lines by *Agrobacterium*-mediated transformation as described previously (Konagaya et al. 2020). We selected the transformed cell lines using 25 and 10 mg l^−1^ kanamycin and meropenem. To avoid discarding the genome-edited portion in a line, we divided each cell mass into four and analyzed the mutation patterns as described below. We regenerated the plantlets from the ET lines as described previously (Konagaya et al. 2020).

### GFP observation

We observed independent line GFP fluorescence as described previously (Nanasato et al. 2021).

### DNA isolation and indel detection in *CjChl I* cells using two-round PCR

For the PCR analysis, we extracted genomic DNA from *C. japonica* cell mass or needles with the “Proteinase K method” according to the instructions of the KOD FX Neo kit (Toyobo). We amplified DNA regions, including the target region, via a two-round PCR approach and performed fragment analysis using a capillary sequencer to detect indel patterns based on the fluorescent PCR (Foley et al. 2009) and Indel Detection by Amplicon Analysis (IDAA) (Yang et al. 2015) approaches with modifications as described below. To analyze the *CjChl I*_t4 region, we used the p#568 and p#465 as well as the T7-Fam_f and p#465 primer pairs to amplify a 215-bp fragment in the 1st and 236-bp in the 2nd PCR rounds, respectively. To analyze the *CjChl I*_t1 and t2 regions, we used the p#569 and p#469 as well as the T7-Fam_f and p#469 primer pairs to amplify a 236-bp fragment in the 1st and 257-bp in the 2nd PCR rounds, respectively. We performed the first-round PCR amplification using the KOD FX Neo DNA polymerase (Toyobo) in a 10-µl PCR mixture with 10 mM α-cyclodextrin for SDS tolerance to PCR (Nakanishi et al. 2022) under the thermal cycling conditions as follows: initial denaturation for 2 min at 94°C, 15 touchdown cycles at 72°C (−1.0°C/cycle) and at 68°C for 30 s, followed by 25 standard cycles of 10 s at 98°C, 30 s at 58°C, and 30 s at 68°C, finishing with a final extension step at 68°C for 7 min. We performed the 2nd PCR round to generate fluorescent PCR products using a 200× diluted first-round PCR product as a template in a 10-µl PCR mixture under the following conditions: initial denaturation for 2 min at 94°C, 12 cycles of 10 s at 98°C, 30 s at 55°C, and 30 s at 68°C, finishing with a final extension step at 68°C for 7 min. We analyzed the fragments using an Applied Biosystems 3500xL Genetic Analyzer and the obtained raw data using GeneMapper (Thermo Fisher Scientific).

### Western blotting

We homogenized the ET samples (100 mg) in liquid nitrogen with a bead homogenizer (Shakemaster, BMS, Tokyo, Japan), then resuspended them in 0.5 ml of extraction buffer containing 20 mM Tris-HCl buffer (pH 8.0), 150 mM NaCl, 1 mM EDTA, 1 mM dithiothreitol, 10% (v/v) glycerol, and 1 mM (p-Amidinophenyl)methanesulfonyl fluoride hydrochloride (APMSF). Next, we centrifuged the homogenate at 15,000×g for 10 min at 4°C, used the supernatant as a protein extract, and analyzed the protein concentrations via the Bradford method using bovine serum albumin as a standard. We separated 10 µg of soluble protein via SDS-PAGE (Mini Protean TGX gel, Bio-Rad, Hercules, CA, USA), then transferred it onto a polyvinylidene difluoride (PVDF) membrane using a wet transfer apparatus (Mini Trans-Blot Electrophoretic Transfer Cell, Bio-Rad) following the manufacturer’s instructions. For Western blotting, we used an anti-Cas9 polyclonal antibody (Guide-it, Cas9 #632607, Takara Bio Inc.; dilution: 1 : 1,000), then incubated the membrane with donkey-derived horseradish peroxidase-conjugated anti-rabbit secondary antibody (Anti-Rabbit IgG, HRP-Linked Whole Ab Donkey, # NA934VS, GE Healthcare, UK; dilution: 1 : 20,000). We detected the antigen–antibody complexes using the ECL Select kit according to the manufacturer’s instructions (Cytiva, Tokyo, Japan) and an optical imaging system (Fusion Solo 4M, Vilber Lourmat, Marne la Vallee, France).

### Statistical analysis

We used Tukey’s multiple comparison tests in R (https://www.r-project.org (Accessed Oct 3, 2024)) for pairwise multiple comparisons of the proportions and considered *p*-values statistically significant at *p*<0.05.

## Results

### SpCas9 codon optimization in *C. japonica*

We calculated the codon usage frequency based on the FLcDNA information in *C. japonica* and created a codon usage table, which we subsequently compared with those of *Arabidopsis thaliana* and *Oryza sativa* (Table 1). The resulting codon usage frequency in *C. japonica* differed from that in *O. sativa* but was comparable to that in *A. thaliana*. Nevertheless, the threonine (Thr)- and alanine (Ala)-related codon choices differed in *C. japonica* and *A. thaliana*: in the latter, the ACU (0.34) and ACA (0.31) codons were used for Thr, while in the former, ACA (0.40) was favored over ACU (0.34). Concerning Ala, GCU (0.43) and GCA (0.27) as well as GCA (0.39) and GCU (0.35) were used in *A. thaliana* and *C. japonica*, respectively. In addition, 7 codons occurred at low frequencies (<10%) in *C. japonica*, e.g., those for Leu (CUA), Ser (UCG), Pro (CCG), Thr (ACG), Ala (GCG), and Arg (CGC and CGA). According to the created codon usage table, we reorganized a codon-optimized Cas9 for *C. japonica*, named ^Cj^SpCas9 (Supplementary Figure S1A), then compared it with ^At^SpCas9 and ^Os^SpCas9 (Supplementary Figure S1B, C) (Mikami et al. 2015), resulting in 82.79% and 77.01% of base-match, respectively (Supplementary Figure S2). Moreover, 77.45% of the bases were identical to those of ^At^SpCas9 and ^Os^SpCas9. To evaluate ^Cj^SpCas9 function, we created a vector incorporating ^At^SpCas9 and codon-optimized ^Os^SpCas9 and introduced it into *C. japonica* to compare and verify the genome editing efficiency in Japanese cedar.

### Genome editing efficiency comparison of the GFP transgene and various SpCas9 genes

To compare the genome editing efficiency of ^Cj^SpCas9 with that of other SpCas9s, we used the single-copy GFP-expressing ET line N4 (Nanasato et al. 2021) for CRISPR/Cas9-mediated genetic modifications in the GFP transgene. We selected a target site in the GFP gene, 199 bp downstream of the translation start site, previously named #2_rev, for the guide RNA gene design as the genome editing efficiency was the highest at this site in the previous trial (Nanasato et al. 2021). We observed a total of 2–22 transgenic ET lines in each SpCas9 (Table 2). Certain lines completely lost the GFP fluorescence, whereas others exhibited it at specific cell mass areas. We considered both lines with the complete or partial loss of GFP fluorescence as GFP knockout lines. The number of lines with modified GFP genes was 1, 2, and 15 for ^At^SpCas9, ^Os^SpCas9, and ^Cj^SpCas9, respectively. While we could have calculated the efficiencies based on the number of transformed lines to the number of GFP knock out lines; however, we obtained only two transformed lines during the ^At^SpCas9-introducing trial. Therefore, we chose not to calculate the efficiency in this experiment.

**Table 2:** Table 2. GFP-related genome editing efficiency comparison on *SpCas9* codon optimization in the N4 line.

Target of *GFP*	Cas9	No. of transformed lines^1^	No. of GFP knock out lines^2^
t2	^At^SpCas9	2	1
	^Os^SpCas9	14	2
	^Cj^SpCas9	22	15

^1^Number of transgenic colonies formed approximately 1–3 months postinfection in the selection medium. Colonies taken up to three times at the same spot were considered the same line. ^2^Number of lines with partially or completely lost GFP fluorescence in the cell mass.

### *CjChl I* gene editing efficiency and fragment analysis-mediated indel detection

**Figure 2:**
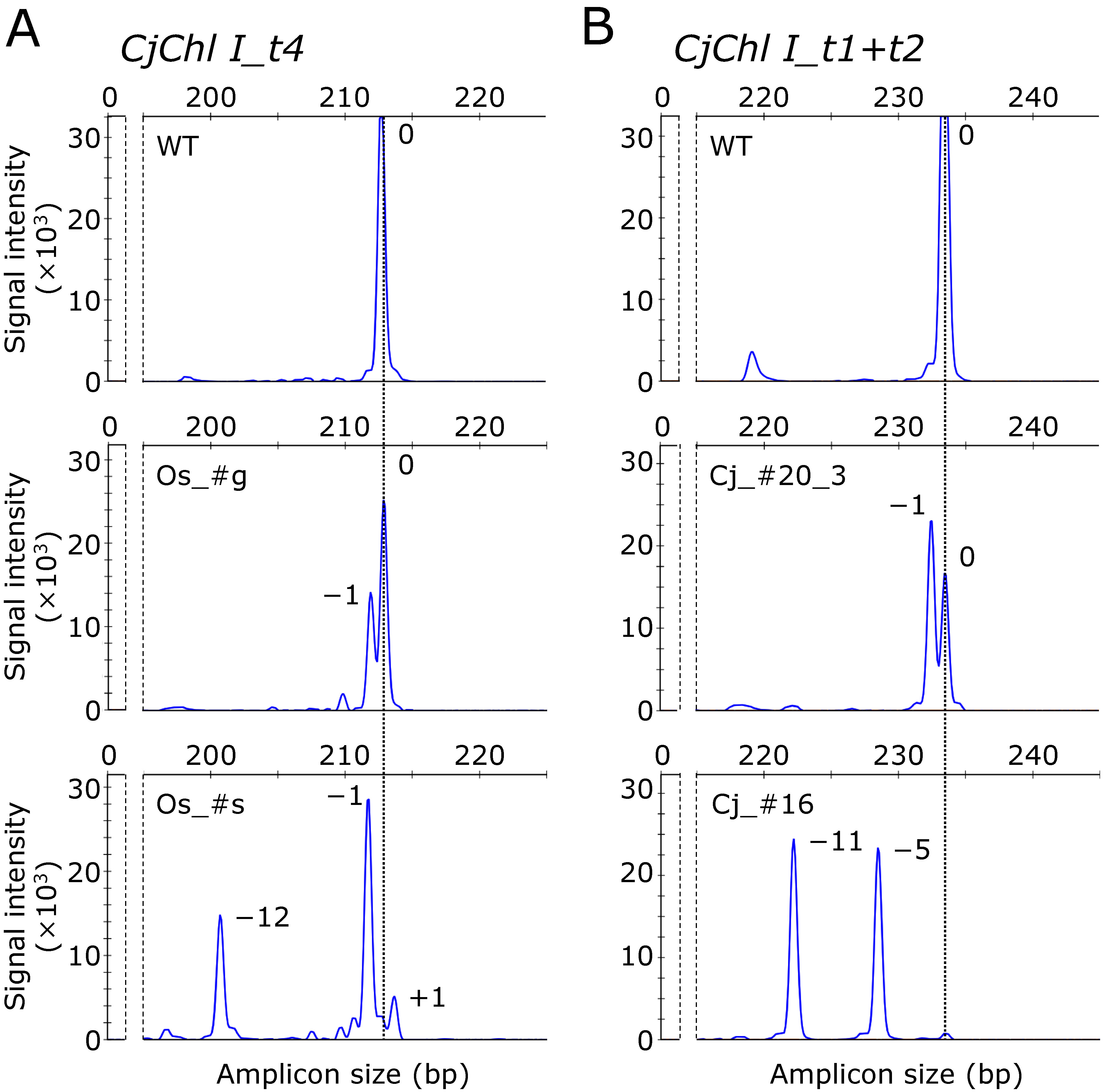
Figure 2. Gene targeting-induced indel detection in *CjChl I* in the ET cell lines using fragment analysis. (A) Mutations around t4 in *CjChl I.* From top to bottom: the amplified unedited-WT amplicon peak, pCRG-^Os^SpCas9_CjChl I_t4-introduced line #g (Os_#g), and pCRG-^Os^SpCas9_CjChl I_t4-introduced line #s (Os_#s), respectively. Os_#g possesses both unmodified and 1-bp deleted alleles. However, Os_#s possesses multiple genome-edited alleles in the cell masses. (B) Mutations around t1 and t2 in *CjChl I*. From top to bottom: the amplified unedited-WT amplicon peak, pCRG-^Cj^SpCas9_CjChl I_t1+t2-introduced line #20-3 (Cj_#20-3) with unmodified and 1 bp deleted alleles, and pCRG-^Cj^SpCas9_CjChl I_t1+t2-introduced line #16 (Cj_#16) with 5 and 11 bp deleted alleles, respectively.

Using the SpCas9 variants, we investigated the genome editing efficiency of endogenous genes in *C. japonica*. Our examined target gene was *CjChl I*, reportedly manifesting a visible albino phenotype upon disruption (Nanasato et al. 2021). We constructed CRISPR/Cas9 vectors, each containing a single gRNA expression cassette for t4 and simultaneously two for t1 and t2 (t1+t2) with each SpCas9 variant (see Figure 1). Following the vector introduction, we obtained 34–70 transgenic lines for each vector. Previously, the heteroduplex mobility assay (HMA) was used to screen putative genome-edited lines (Nanasato et al. 2021). However, the short (<3 bp) indel detection capacity of HMA is limited due to its resolution, despite the genome editing-induced short indel prevalence in *C. japonica* (Nanasato et al. 2021). To overcome this limitation, we applied IDAA, a fragment analysis method with 1-bp indel detection precision, to screen putative genome-edited lines. In t4-targeting *CjChl I* editing, we detected an approximately 213-bp unmodified amplicon in the wild type (WT), whereas an amplicon with a 1-bp deletion compared with the WT as well as an unmodified amplicon in the pCRG-^Os^SpCas9_CjChl I_t4-introduced line #g (Os_#g line), and multiple amplicons with 1-bp insertions and 1- and 12-bp deletions in the pCRG-^Os^SpCas9_CjChl I_t4-introduced line #s (Os_#s line) (Figure 2A). Similarly, when targeting t1+t2, we observed only unmodified bands in the WT, whereas in the pCRG-^Cj^SpCas9_CjChl I_t1+t2-introduced line #20-3 (Cj_#20-3 line), a 1-bp loss along with unmodified bands from the WT. In the pCRG-^Cj^SpCas9_CjChl I_t1+t2-introduced line #16 (Cj_#16 line), we registered no WT-derived bands at all, and amplified bands lacking 5, and 11 bp (Figure 2B). The genome editing efficiencies ranged between 20.6–78.4% and 35.5–65.7% in t4 and t1+t2, respectively (Table 3). In the case of both targets, the genome editing efficiency was the highest when ^Cj^SpCas9 was introduced.

**Table 3:** Table 3. Genome editing efficiency comparison of *CjChl I* on *SpCas9* codon optimization in the transgenic ET lines.

Target in *CjChl I*	Cas9	No. of transformed lines^1^ (A)	No. of IDAA-positive lines^2^ (B)	Efficiency^3^ (%)
t4	^At^SpCas9	34	7	20.6^b^
	^Os^SpCas9	50	14	28.0^b^
	^Cj^SpCas9	51	40	78.4^a^
t1+t2	^At^SpCas9	31	11	35.5^d^
	^Os^SpCas9	35	14	40.0^d^
	^Cj^SpCas9	70	46	65.7^c^

^1^Number of transgenic colonies formed approximately 1–3 months postinfection in the selection medium. Colonies taken up to three times at the same spot were considered the same line. ^2^Number of lines with insertions or deletions in the target site-containing amplicons detected by fragment analysis (the Indel Detection by Amplicon Analysis, or IDAA). ^3^(B/A)×100. Pairwise multiple comparison of proportions performed by Tukey’s multiple comparison test. Proportions with significant differences were labeled with different letters (*p*<0.05). This table summarizes the total results of three independent experiments.

### Codon variation effect on Cas9 protein accumulation

**Figure 3:**
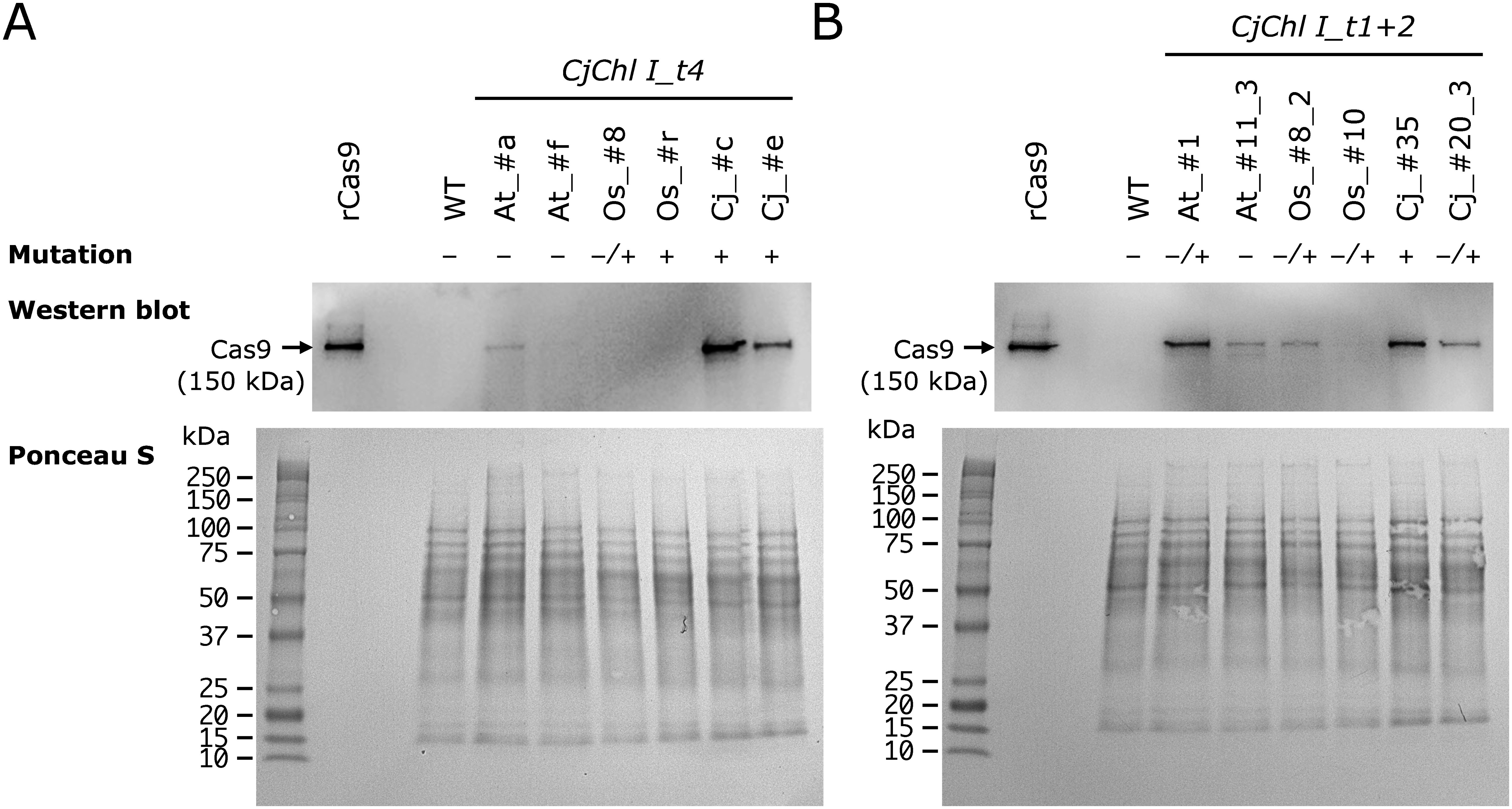
Figure 3. Western blot analysis of SpCas9 proteins in ET cells of transgenic *C. japonica* expressing CRISPR/Cas9. A 10 µg aliquot of soluble proteins was loaded per lane. Five ng of recombinant SpCas9 (rCas9) was applied on the left as a positive control. Ponceau S staining of the filters was performed to ensure approximately equal loading of protein. Mutation: “−”, unmodified amplicon; “−/+”, unmodified amplicon along with indel(s); “+”, amplicon(s) with indel(s) only.

Genome editing efficiency was improved upon SpCas9 codon optimization. To determine whether this effect was due to increased SpCas9 protein accumulation, we analyzed SpCas9 protein levels in randomly selected ET lines (Supplementary Figure S3) using Western blot analysis. We observed Cas9 protein accumulation in the case of each of the two cell lines containing the introduced different CRISPR/Cas9 vectors, and detected SpCas9 protein accumulation at 150 kDa (Figure 3). As expected, such accumulation was higher in the ^Cj^SpCas9 vector-containing lines, especially in that carrying *CjChl I_t4*. Cas9 protein levels were similar between ^At^SpCas9 and ^Os^SpCas9.

### Phenotypical genome editing effects of *CjChl I* in regenerated plantlets

**Figure 4:**
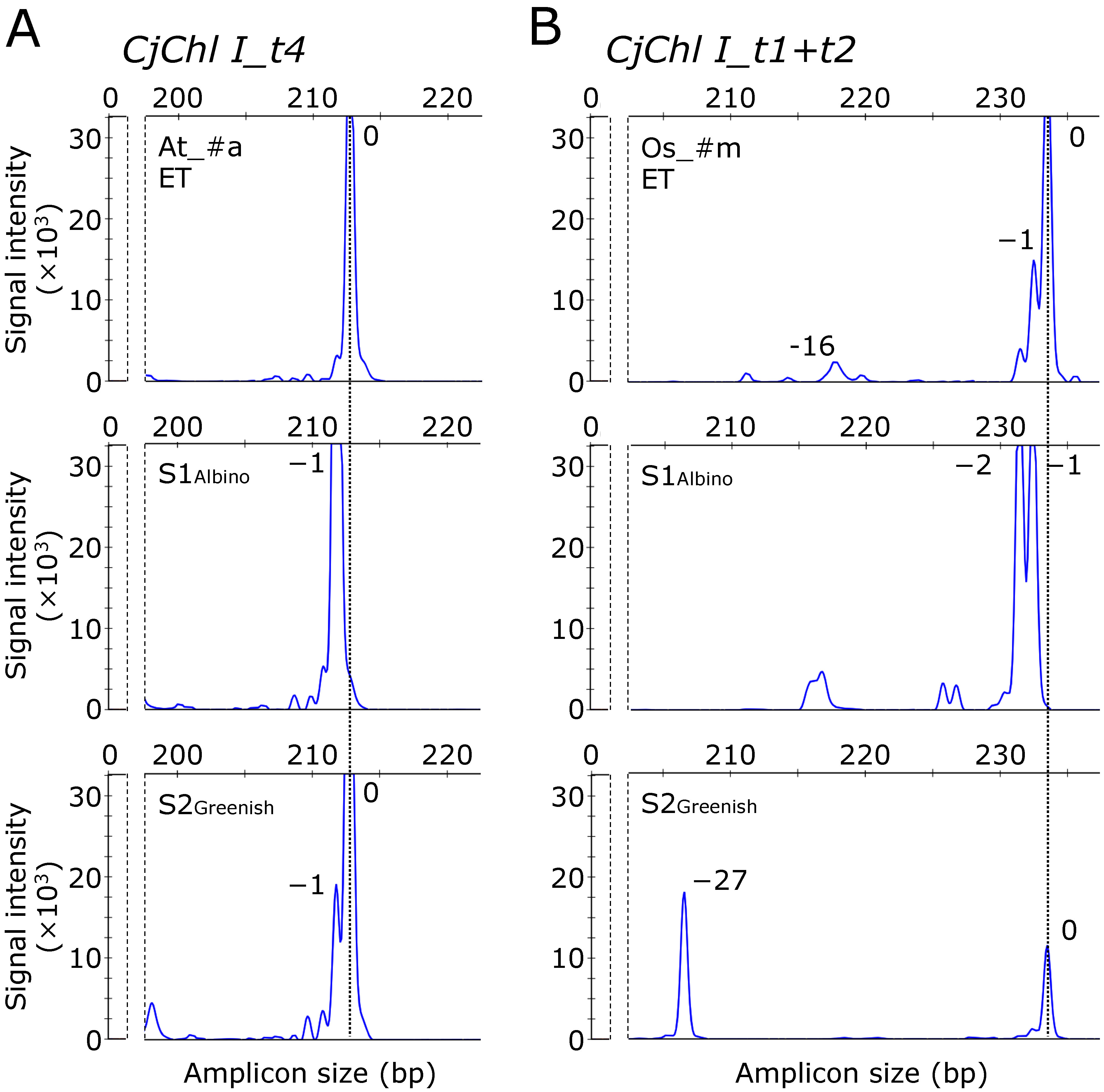
Figure 4. Novel mutations in somatic embryogenesis. (A) Mutations around t4 in *CjChl I* in the pCRG-^At^SpCas9_CjChl I_t4-carrier line #a (At_#a.) From top to bottom: amplified amplicon peaks from the ET cell masses and 2 generated seedlings (S1 and S2.) Only an unmodified WT amplicon peak was detected in the At_#a cell masses. However, S1 displayed novel 1-bp deleted alleles with an albino phenotype. S2 exhibited both unmodified and novel 1-bp deleted alleles with a greenish phenotype. (B) Mutations around t1 and t2 in *CjChl I* in pCRG-^Os^SpCas9_CjChl I_t1+t2-introduced line #m (Os_#m.) From top to bottom: amplified amplicon peaks from the ET cell masses and 2 generated seedlings (S1 and S2.) The Os_#m cell masses contained unmodified and 1-bp deleted alleles. S1 displayed 1-bp and novel 2-bp deleted alleles and an albino phenotype. However, S2 exhibited unmodified and novel 27-bp deleted alleles with a greenish phenotype.

**Figure 5:**
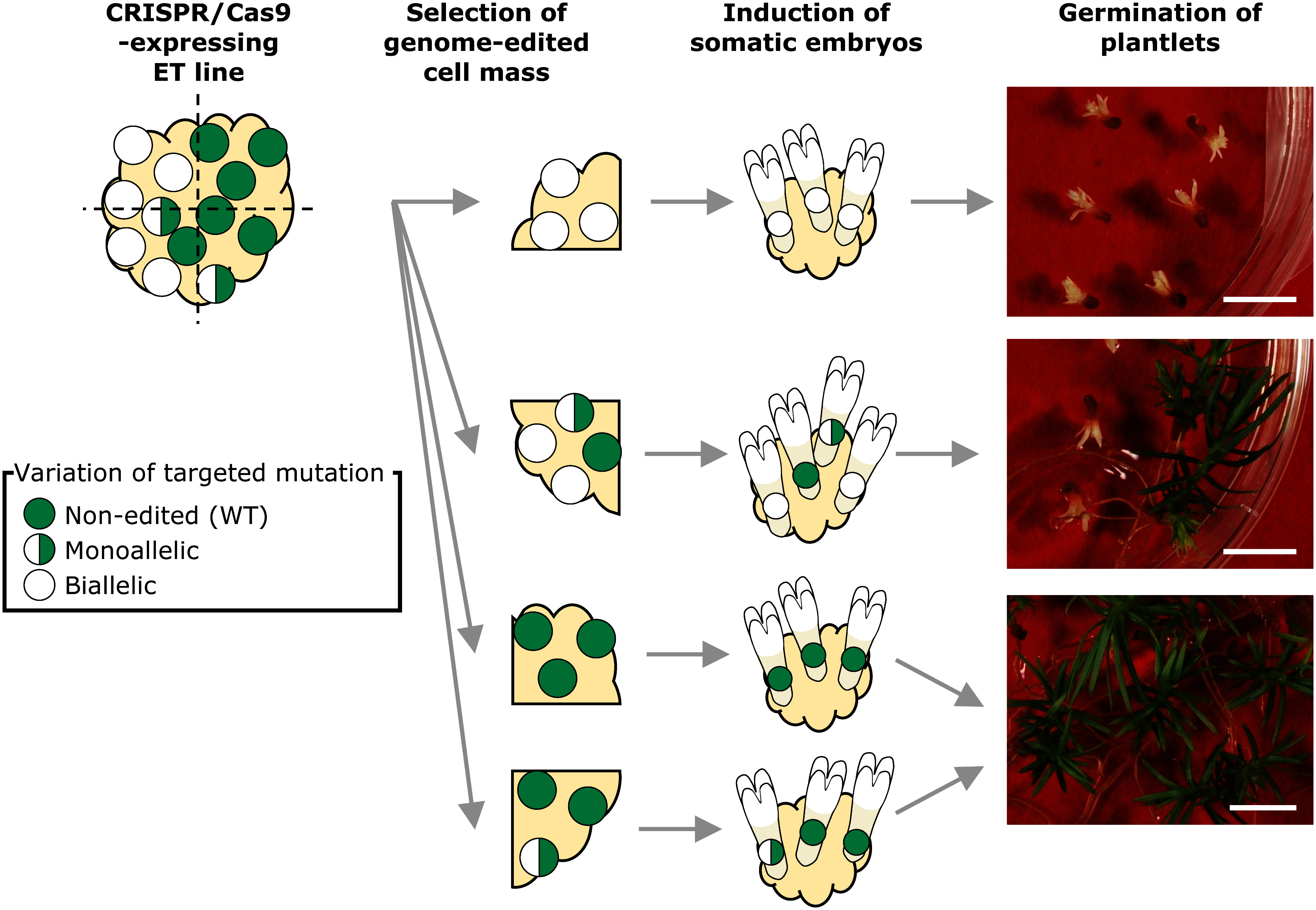
Figure 5. Schematic diagram of plantlet production using targeted *CjChl I* mutation. An ET line containing a CRISPR/Cas9 expression cassette often has multiple mutation patterns in the cell mass. The cell mass was divided into 2–4 parts so that the cell masses with high-rate genome editing could be selected. Albino plantlets were generated from cell masses with biallelic mutations. This figure omits the descriptions of chimera plantlets resulting from new target mutations induced during the development of ET cells into plantlets. Scale bar: 1 cm.

To assess the phenotypical effects of *CjChl I* genome editing, we induced plantlet regeneration via somatic embryogenesis, observed the color of these plantlets, and performed IDAA analysis in each line. For the genome-edited lines targeting t4 in *CjChl I*, we selected and regenerated 26, 31, and 37 ^At^SpCas9-, ^Os^SpCas9-, and ^Cj^SpCas9-carrier lines, respectively (Table 4). We observed albino phenotypes in the IDAA-positive lines at proportions of 80.0%, 63.6%, and 74.1%, respectively. However, we also obtained albino individuals (i.e., 25%, 12.5%, and 25%, respectively) from certain ET lines, in which we could not detect indels during the IDAA analysis. Taken together, the percentages of albino individuals in the regenerated lines were 38.1%, 33.3%, and 67.7%, respectively. For the t1+t2-targeting genome-edited lines in *CjChl I*, we selected and regenerated 26, 31, and 60 ^At^SpCas9-, ^Os^SpCas9-, and ^Cj^SpCas9-carrier lines, respectively (Table 4), with observed albino phenotypes in the IDAA-positive lines at proportions of 16.6%, 58.3%, 63.6%, respectively. However, we obtained albino individuals also from certain ET lines without any detected indel during the IDAA analysis (i.e., 0%, 7.7%, and 6.3%, respectively). Taken together, the albino individual proportions in the regenerated lines were 9.1%, 32.0%, and 44.9%. For both targets, we achieved the highest genome editing efficiency in the case of ^Cj^SpCas9. In this study, we identified multiple mutation patterns in the plantlets that were not detected in the ET lines (Supplementary Table S3A, B). For example, in the t4 At_#a line, we registered a 1-bp deletion in Seedling 1, and 0 and 1 bp deletions in Seedling 2, regenerated from ET, even though we observed no mutation in the ET source. The phenotypes also reflected the fragment analysis results (Figure 4A). When targeting t1+t2, the Os_#m ET line contained 0 and 1 bp deletions, whereas Seedling 1 carried a 2-bp deletion beyond the 1-bp deletion in the ET line. In Seedling 2, we newly identified 27-bp deletions beyond the wild type deletions in the ET line. These phenotypes were white and green, reflecting the fragment analysis results (Figures 4B, 5).

**Table 4:** Table 4. *CjChl I* genome-edited plantlet phenotypes with various *SpCas9* genes.

Target in *CjChl I*	Cas9	No. of ET lines used in this experiment	Indel(s) in ET lines	No. of ET lines	No. of germinated lines	No. of albino lines	Efficiency (%)^1^
t4	^At^SpCas9	26	+	6	5	4	38.1^a^
			−	20	16	4	
	^Os^SpCas9	31	+	12	11	7	33.3^a^
			−	19	16	2	
	^Cj^SpCas9	37	+	31	27	20	67.7^b^
			−	6	4	1	
t1+t2	^At^SpCas9	26	+	10	6	1	9.1^c^
			−	16	5	0	
	^Os^SpCas9	31	+	13	12	7	32.0^c^
			−	18	13	1	
	^Cj^SpCas9	60	+	40	33	21	44.9^c^
			−	20	16	1	

^1^Efficiency: (No. of albino lines/No. of germinated lines)×100. Pairwise multiple comparison of proportions performed by Tukey’s multiple comparison test. Proportions with significant differences were labeled with different letters (*p*<0.05). This table summarizes the total results of three independent experiments.

## Discussion

In a previous study, we isolated U6 promoters from *C. japonica* to improve the CRISPR/Cas9 expression vector and compared their activity with those from *Arabidopsis thaliana* and *Oryza sativa* (Nanasato et al. 2021). The results demonstrated similar U6 promoter activities in *C. japonica*, *A. thaliana*, and *O. sativa*. However, since ^At^SpCas9 was always used for Cas9 protein expression, the Cas9 protein expression level was predictably low, suggesting that the DNA cleavage activity was potentially limited by the Cas9 protein amount rather than the gRNA expression level. We observed increased Cas9 protein expression levels upon codon optimization (Figure 3) and improved genome editing efficiency in terms of target gene biallelic disruption rates (Table 4, Supplementary Table S3a, b). In particular, when targeting t4 in *CjChl I*, the biallelic mutant number was the highest when ^Cj^SpCas9 was used (Table 4). Unlike when targeting t4, in the case of targeting t1+t2 in *CjChl I*, the number of individuals with biallelic mutations increased upon ^Cj^SpCas9 application, although the difference was not statistically significant compared with that when using ^At^SpCas9 or ^Os^SpCas9, potentially due to the tandem gRNA application-related genome editing efficiency improvement (Joberty et al. 2020).

The IDAA analysis we used in this study could discriminate single-base indels in a short time. This is a useful method that provides a one-step higher resolution than previously reported approaches (e.g., HMA and T7 endonuclease assays). When targeting t4 in *CjChl I*, certain individuals displayed a greenish phenotype despite carrying mutations (Supplementary Table S3), several of which were multiples of three. An indel with multiples of three is not a frameshift mutation that does not lead to gene disruption. Therefore, they must be excluded as gene disruption lines. Excluding mutants with multiples of three would be useful in the screening process. As the results of this study demonstrated, IDAA is an advantageous method for analyzing the presence or absence of not only indels but also of frameshifts, since indel lengths could be analyzed. In-advance PCR cycle number optimization is important as an excessive amount of PCR cycles would yield extra bands, making result interpretation difficult. In the original study on IDAA, Yang et al. (2015) used a method with three primers, one of which was an FAM-modified T7 primer. However, in this study, we performed a two-round PCR to increase PCR efficiency. The disadvantage of the two-round PCR is the more troublesome and time-consuming process. While it is expensive, using FAM-labeled specific primer pairs for target region amplification allows for shortening the process into a single step. However, the IDAA analysis is not adapted for detecting long deletions around the target region due to the primer sequence deletion(s), which is a major disadvantage of short-read-based analyses. In such cases, next-generation long-read sequencing, such as Oxford nanopore sequencing, could be very useful to complement fragment analysis results (Sato et al. 2024).

In the seedlings, we detected indel patterns other than those observed in the ET cells (Figure 4, Supplementary Table S3). We previously reported similar results in the case of genome editing GFP-expressing *C. japonica* cells (Nanasato et al. 2021). We hypothesized that we obtained this result due to ongoing Cas9-mediated DNA cleavage related to continuous Cas9 protein expression in the cells (Nanasato et al. 2021). Our results in the present study support this hypothesis (Figure 4, Supplementary Table S3). Therefore, we could reasonably assume that genome editing-mediated gene modification efficiency increases with extended culture periods. However, the differentiation ability from embryogenic tissues to somatic embryos is often lost within one year during repeated subculture (Konagaya et al. 2020). Therefore, selecting lines that are expectably as completely disrupted around a target site as possible within a short time would be important for fully gene-disrupted plantlet generation. Complete control over the genome editing approach would require next-generation genome editing approaches (e.g., base editors and Prime Editing) (Komor et al. 2016; Nishida et al. 2016; Zong et al. 2022).

Recently, male-sterile *C. japonica* lines have been created using the CRISPR/Cas9 system (Nishiguchi et al. 2023), representing the beginning of *C. japonica* molecular breeding via genome editing. Using codon-optimized SpCas9, we currently aim at developing several male-sterile *C. japonica* lines by modifying male strobilus development-related genes and establishing a precise genome editing technique using a base editor system. We hope that the hereby presented study would contribute to accelerating research on other important conifers, such as hinoki cypress (*C. obtusa*) and Japanese larch (*L. kaempferi*).

## Abbreviations

CRISPR/Cas9: clustered regularly interspaced short palindromic repeats (CRISPR)/CRISPR-associated protein 9
SpCas9: *Streptococcus pyogenes* Cas9

## Description of Supplementary Files

Supplementary Figure S1. Sequence information of ^Cj^SpCas9, ^At^SpCas9, and ^Os^SpCas9.

Supplementary Figure S2. Amino acid sequence of SpCas9 and sequence alignment of the Cas9 genes from ^Cj^SpCas9, ^At^SpCas9, and ^Os^SpCas9.

Supplementary Figure S3. Indel patterns of ET cell lines induced by gene targeting.

Supplementary Table S1. Information about coding regions used to calculate codon frequency.

Supplementary Table S2. List of oligonucleotides used in this study.

Supplementary Table S3. Phenotypes of seedlings genome-edited at t4 and t1+t2 in *CjChl I* using various SpCas9 variants.
