# Supplementary material for: Improvement of genome editing efficiency by Cas9 codon optimization in Japanese cedar (Cryptomeria japonica D. Don)
Source: Plant Biotechnol (Tokyo). 2024 Dec 25;41(4):335–44. doi: 10.5511/plantbiotechnology.24.0709a (PMC11897717; doi:10.5511/plantbiotechnology.24.0709a)
Supplement: Supplementary Data [file plantbiotechnology-41-4-24.0709a-s001.pdf]

ATGGACAAGAAGTACAGCATCGGCCTGGATATCGGCACAAATTCTGTTGGCTGGGCCGTGATTACCGACGAGTATAAGGTTCCCAGCAAGAAGT
TCAAGGTCCTGGGCAACACAGACAGGCACAGCATTAAGAAGAATCTGATTGGCGCCCTGCTGTTCGATTCTGGTGAAACAGCAGAGGCAACAAG
GCTGAAGAGGACAGCTAGAAGAAGGTACACCAGGCGTAAGAACAGGATCTGCTACCTGCAAGAGATCTTCAGCAACGAGATGGCCAAGGTGGAC
GACAGCTTTTTCCATAGGCTGGAAGAGAGCTTCCTGGTCGAAGAGGATAAGAAGCACGAGAGGCATCCTATCTTCGGCAACATTGTGGATGAGG
TGGCATACCACGAGAAGTACCCTACAATCTACCACCTGAGGAAGAAGCTGGTTGACAGCACAGATAAGGCAGACCTGAGGCTGATCTATCTGGC
ACTGGCACACATGATTAAGTTCAGGGGCCACTTTCTGATCGAGGGCGATCTGAATCCCGACAACTCTGATGTGGACAAGCTGTTCATTCAGCTG
GTGCAGACATACAACCAGCTGTTCGAAGAGAACCCCATCAACGCATCTGGTGTGGATGCAAAGGCAATTCTGTCTGCCAGGCTGAGCAAGTCAA
GAAGGTTGGAGAACCTGATTGCACAGTTGCCTGGCGAGAAGAAGAATGGCCTGTTCGGAAACCTCATTGCACTGTCTTTGGGCCTGACACCTAA
CTTCAAGAGCAACTTCGATCTGGCCGAGGATGCTAAGTTGCAGCTGTCTAAGGATACCTACGATGACGACCTGGATAACCTGCTGGCACAGATT
GGAGATCAGTACGCAGATCTGTTCCTGGCAGCAAAGAACCTGTCTGATGCCATCCTGCTGAGCGATATTCTGAGGGTGAACACCGAGATTACAA
AGGCCCCTCTGAGCGCCTCTATGATCAAGAGATACGATGAGCACCACCAGGATCTGACATTGCTGAAGGCATTGGTTAGGCAGCAGCTGCCAGA
GAAGTACAAAGAGATTTTCTTCGATCAGAGCAAGAACGGCTACGCCGGCTATATTGATGGTGGTGCATCTCAAGAAGAGTTCTACAAGTTCATC
AAGCCCATCCTCGAGAAGATGGATGGCACAGAGGAATTGCTGGTGAAGCTGAACAGAGAGGACTTGTTGAGGAAGCAGAGGACCTTCGATAACG
GCAGCATTCCTCATCAGATTCATCTGGGAGAGCTGCACGCAATCTTGAGAAGGCAAGAGGACTTCTACCCATTCCTGAAGGACAACCGTGAGAA
GATAGAGAAGATCCTGACCTTCAGGATCCCCTATTATGTGGGACCACTGGCAAGGGGCAATTCTAGGTTTGCTTGGATGACCCGTAAGAGCGAA
GAGACAATTACCCCTTGGAACTTCGAAGAGGTGGTGGATAAGGGCGCATCTGCCCAGTCTTTTATTGAGAGGATGACCAACTTCGACAAGAACC
TGCCTAACGAGAAGGTGCTGCCAAAGCACTCTCTGCTGTACGAGTACTTCACCGTGTACAACGAGCTGACCAAAGTGAAGTATGTGACCGAGGG
CATGAGAAAGCCTGCATTTTTGTCTGGGGAGCAGAAGAAGGCCATCGTCGATTTGCTGTTCAAGACCAACAGAAAGGTGACCGTGAAGCAGCTG
AAAGAGGACTACTTCAAGAAAATCGAGTGCTTCGACAGCGTCGAGATCTCTGGTGTTGAGGATAGGTTCAATGCCAGCCTGGGCACATATCACG
ACCTGCTCAAGATCATCAAGGACAAGGACTTTCTGGACAACGAGGAAAACGAGGACATCTTGGAGGATATCGTGCTGACACTGACCCTCTTCGA
GGATAGAGAGATGATCGAGGAAAGGCTCAAGACATACGCACACCTGTTCGACGACAAGGTGATGAAGCAGTTGAAGCGTAGAAGGTATACAGGC
TGGGGCAGACTGTCACGTAAGCTGATTAACGGCATCAGGGATAAGCAGAGCGGCAAGACCATTCTCGACTTTCTGAAGTCTGATGGCTTCGCCA
ACCGTAACTTCATGCAGCTGATCCACGATGACAGCCTCACCTTCAAAGAGGATATCCAGAAGGCACAGGTTAGCGGACAAGGCGATTCTCTGCA
TGAGCACATTGCAAATCTGGCAGGCAGCCCTGCTATTAAGAAGGGCATTCTGCAGACAGTGAAGGTCGTGGATGAGTTGGTTAAGGTGATGGGC
AGACACAAGCCTGAGAACATCGTGATTGAGATGGCACGTGAGAACCAGACAACTCAGAAGGGCCAGAAGAACTCCAGGGAACGTATGAAGAGGA
TTGAAGAGGGCATCAAAGAGCTGGGCTCCCAGATTTTGAAAGAGCACCCTGTTGAGAACACCCAGCTGCAGAACGAGAAGCTGTATCTGTACTA
CTTGCAGAACGGCAGGGACATGTATGTGGATCAAGAGCTGGATATCAACAGGCTGTCCGATTACGACGTGGACCATATTGTGCCTCAGAGCTTC
TTGAAGGACGACTCCATCGATAACAAGGTGCTGACCAGGTCCGATAAGAACAGAGGCAAGAGCGATAATGTGCCCTCCGAAGAGGTTGTGAAGA
AGATGAAGAACTACTGGCGTCAGCTCCTGAACGCCAAGTTGATTACTCAGAGGAAGTTCGACAACCTGACCAAGGCAGAAAGAGGCGGATTGAG
CGAATTGGATAAGGCCGGCTTTATTAAGAGGCAGCTGGTGGAAACAAGGCAGATCACAAAACACGTGGCCCAGATTCTGGACTCCAGGATGAAC
ACAAAGTACGACGAGAATGACAAGCTGATCCGTGAGGTGAAGGTCATCACCCTGAAGTCTAAGCTGGTGAGCGACTTCCGTAAGGACTTCCAGT
TCTATAAGGTGCGAGAGATCAACAACTACCACCACGCACATGATGCCTACCTGAATGCAGTTGTGGGCACAGCACTGATCAAGAAGTATCCCAA
GCTCGAGAGCGAGTTCGTGTACGGTGATTACAAGGTGTACGACGTGAGGAAGATGATCGCCAAGAGCGAGCAAGAGATTGGCAAGGCAACAGCC
AAGTACTTCTTCTACAGCAACATCATGAATTTCTTCAAGACCGAGATCACCCTGGCCAACGGCGAAATTAGGAAGAGGCCTTTGATTGAGACAA
ACGGCGAGACAGGTGAGATCGTGTGGGATAAGGGTAGAGATTTCGCCACAGTGCGTAAGGTGCTGTCTATGCCTCAGGTGAACATTGTGAAAAA
GACCGAGGTTCAGACCGGCGGCTTCAGCAAAGAGTCTATTCTGCCTAAGAGGAACAGCGATAAGCTGATCGCCAGAAAGAAGGATTGGGACCCT
AAGAAGTATGGCGGCTTCGATTCTCCTACAGTGGCATATTCTGTGCTGGTGGTGGCAAAGGTTGAGAAGGGGAAGTCAAAGAAGCTGAAGTCCG
TCAAAGAGTTGCTGGGCATCACAATCATGGAAAGGTCCAGCTTCGAGAAGAATCCTATCGACTTCTTGGAGGCCAAGGGCTACAAAGAGGTGAA
GAAGGACCTCATCATCAAGCTGCCCAAGTACTCACTGTTCGAGTTGGAGAATGGCCGTAAGAGGATGTTGGCATCTGCTGGCGAATTGCAGAAG
GGAAACGAATTGGCACTGCCCTCCAAGTACGTGAACTTCCTGTATCTGGCAAGCCACTACGAGAAGTTGAAGGGCTCTCCTGAGGATAACGAGC
AGAAGCAGCTTTTCGTCGAGCAGCATAAGCACTACCTGGACGAGATTATCGAGCAGATCAGCGAGTTCAGCAAGAGGGTGATACTGGCAGATGC
AAACCTGGACAAGGTGTTGAGCGCATACAACAAGCACAGGGACAAGCCTATTAGAGAGCAGGCCGAGAACATCATCCACCTGTTCACACTGACA
AATCTGGGTGCTCCTGCAGCCTTCAAGTATTTCGATACCACCATCGACCGAAAGAGGTACACCTCCACAAAAGAGGTGTTGGACGCCACACTGA
TCCACCAGTCTATTACAGGACTGTACGAGACAAGGATCGACCTGTCTCAACTTGGAGGTGATAGTAGGGCCGATCCTAAGAAGAAGAGGAAGGT
TTAG

Supplementary Figure S1A. Sequence information of ^{Cj}SpCas9, codon optimized for *Cryptomeria japonica*. Underlined is the SV40 nuclear localization signal (NLS).

```
ATGGATAAGAAGTACTCTATCGGACTCGATATCGGAACTAACTCTGTGGGATGGGCTGTGATCACCGATGAGTACAAGGTGCCATCTAAGAAGT
TCAAGGTTCTCGGAAACACCGATAGGCACTCTATCAAGAAAAACCTTATCGGTGCTCTCCTCTTCGATTCTGGTGAAACTGCTGAGGCTACCAG
ACTCAAGAGAACCGCTAGAAGAAGGTACACCAGAAGAAAGAACAGGATCTGCTACCTCCAAGAGATCTTCTCTAACGAGATGGCTAAAGTGGAT
GATTCATTCTTCCACAGGCTCGAAGAGTCATTCCTCGTGGAAGAAGATAAGAAGCACGAGAGGCACCCTATCTTCGGAAACATCGTTGATGAGG
TGGCATACCACGAGAAGTACCCTACTATCTACCACCTCAGAAAGAAGCTCGTTGATTCTACTGATAAGGCTGATCTCAGGCTCATCTACCTCGC
TCTCGCTCACATGATCAAGTTCAGAGGACACTTCCTCATCGAGGGTGATCTCAACCCTGATAACTCTGATGTGGATAAGTTGTTCATCCAGCTC
GTGCAGACCTACAACCAGCTTTTCGAAGAGAACCCTATCAACGCTTCAGGTGTGGATGCTAAGGCTATCCTCTCTGCTAGGCTCTCTAAGTCAA
GAAGGCTTGAGAACCTCATTGCTCAGCTCCCTGGTGAGAAGAAGAACGGACTTTTCGGAAACTTGATCGCTCTCTCTCTCGGACTCACCCCTAA
CTTCAAGTCTAACTTCGATCTCGCTGAGGATGCAAAGCTCCAGCTCTCAAAGGATACCTACGATGATGATCTCGATAACCTCCTCGCTCAGATC
GGAGATCAGTACGCTGATTTGTTCCTCGCTGCTAAGAACCTCTCTGATGCTATCCTCCTCAGTGATATCCTCAGAGTGAACACCGAGATCACCA
AGGCTCCACTCTCAGCTTCTATGATCAAGAGATACGATGAGCACCACCAGGATCTCACACTTCTCAAGGCTCTTGTTAGACAGCAGCTCCCAGA
GAAGTACAAAGAGATTTTCTTCGATCAGTCTAAGAACGGATACGCTGGTTACATCGATGGTGGTGCATCTCAAGAAGAGTTCTACAAGTTCATC
AAGCCTATCCTCGAGAAGATGGATGGAACCGAGGAACTCCTCGTGAAGCTCAATAGAGAGGATCTTCTCAGAAAGCAGAGGACCTTCGATAACG
GATCTATCCCTCATCAGATCCACCTCGGAGAGTTGCACGCTATCCTTAGAAGGCAAGAGGATTTCTACCCATTCCTCAAGGATAACAGGGAAAA
GATTGAGAAGATTCTCACCTTCAGAATCCCTTACTACGTGGGACCTCTCGCTAGAGGAAACTCAAGATTCGCTTGGATGACCAGAAAGTCTGAG
GAAACCATCACCCCTTGGAACTTCGAAGAGGTGGTGGATAAGGGTGCTAGTGCTCAGTCTTTCATCGAGAGGATGACCAACTTCGATAAGAACC
TTCCAAACGAGAAGGTGCTCCCTAAGCACTCTTTGCTCTACGAGTACTTCACCGTGTACAACGAGTTGACCAAGGTTAAGTACGTGACCGAGGG
AATGAGGAAGCCTGCTTTTTTGTCAGGTGAGCAAAAGAAGGCTATCGTTGATCTCTTGTTCAAGACCAACAGAAAGGTGACCGTGAAGCAGCTC
AAAGAGGATTACTTCAAGAAAATCGAGTGCTTCGATTCAGTTGAGATTTCTGGTGTTGAGGATAGGTTCAACGCATCTCTCGGAACCTACCACG
ATCTCCTCAAGATCATTAAGGATAAGGATTTCTTGGATAACGAGGAAAACGAGGATATCTTGGAGGATATCGTTCTTACCCTCACCCTCTTTGA
AGATAGAGAGATGATTGAAGAAAGGCTCAAGACCTACGCTCATCTCTTCGATGATAAGGTGATGAAGCAGTTGAAGAGAAGAAGATACACTGGT
TGGGGAAGGCTCTCAAGAAAGCTCATTAACGGAATCAGGGATAAGCAGTCTGGAAAGACAATCCTTGATTTCCTCAAGTCTGATGGATTCGCTA
ACAGAAACTTCATGCAGCTCATCCACGATGATTCTCTCACCTTTAAAGAGGATATCCAGAAGGCTCAGGTTTCAGGACAGGGTGATAGTCTCCA
TGAGCATATCGCTAACCTCGCTGGATCTCCTGCAATCAAGAAGGGAATCCTCCAGACTGTGAAGGTTGTGGATGAGTTGGTGAAGGTGATGGGA
AGGCATAAGCCTGAGAACATCGTGATCGAAATGGCTAGAGAGAACCAGACCACTCAGAAGGGACAGAAGAACTCTAGGGAAAGGATGAAGAGGA
TCGAGGAAGGTATCAAAGAGCTTGGATCTCAGATCCTCAAAGAGCACCCTGTTGAGAACACTCAGCTCCAGAATGAGAAGCTCTACCTCTACTA
CCTCCAGAACGGAAGGGATATGTATGTGGATCAAGAGTTGGATATCAACAGGCTCTCTGATTACGATGTTGATCATATCGTGCCACAGTCATTC
TTGAAGGATGATTCTATCGATAACAAGGTGCTCACCAGGTCTGATAAGAACAGGGGTAAGAGTGATAACGTGCCAAGTGAAGAGGTTGTGAAGA
AAATGAAGAACTATTGGAGGCAGCTCCTCAACGCTAAGCTCATCACTCAGAGAAAGTTCGATAACTTGACTAAGGCTGAGAGGGGAGGACTCTC
TGAATTGGATAAGGCAGGATTCATCAAGAGGCAGCTTGTGGAAACCAGGCAGATCACTAAGCACGTTGCACAGATCCTCGATTCTAGGATGAAC
ACCAAGTACGATGAGAACGATAAGTTGATCAGGGAAGTGAAGGTTATCACCCTCAAGTCAAAGCTCGTGTCTGATTTCAGAAAGGATTTCCAAT
TCTACAAGGTGAGGGAAATCAACAACTACCACCACGCTCACGATGCTTACCTTAACGCTGTTGTTGGAACCGCTCTCATCAAGAAGTATCCTAA
GCTCGAGTCAGAGTTCGTGTACGGTGATTACAAGGTGTACGATGTGAGGAAGATGATCGCTAAGTCTGAGCAAGAGATCGGAAAGGCTACCGCT
AAGTATTTCTTCTACTCTAACATCATGAATTTCTTCAAGACCGAGATTACCCTCGCTAACGGTGAGATCAGAAAGAGGCCACTCATCGAGACAA
ACGGTGAAACAGGTGAGATCGTGTGGGATAAGGGAAGGGATTTCGCTACCGTTAGAAAGGTGCTCTCTATGCCACAGGTGAACATCGTTAAGAA
AACCGAGGTGCAGACCGGTGGATTCTCTAAAGAGTCTATCCTCCCTAAGAGGAACTCTGATAAGCTCATTGCTAGGAAGAAGGATTGGGACCCT
AAGAAATACGGTGGTTTCGATTCTCCTACCGTGGCTTACTCTGTTCTCGTTGTGGCTAAGGTTGAGAAGGGAAAGAGTAAGAAGCTCAAGTCTG
TTAAGGAACTTCTCGGAATCACTATCATGGAAAGGTCATCTTTCGAGAAGAACCCAATCGATTTCCTCGAGGCTAAGGGATACAAAGAGGTTAA
GAAGGATCTCATCATCAAGCTCCCAAAGTACTCACTCTTCGAACTCGAGAACGGTAGAAAGAGGATGCTCGCTTCTGCTGGTGAGCTTCAAAAG
GGAAACGAGCTTGCTCTCCCATCTAAGTACGTTAACTTTCTTTACCTCGCTTCTCACTACGAGAAGTTGAAGGGATCTCCAGAAGATAACGAGC
AGAAGCAACTTTTCGTTGAGCAGCACAAGCACTACTTGGATGAGATCATCGAGCAGATCTCTGAGTTCTCTAAAAGGGTGATCCTCGCTGATGC
AAACCTCGATAAGGTGTTGTCTGCTTACAACAAGCACAGAGATAAGCCTATCAGGGAACAGGCAGAGAACATCATCCATCTCTTCACCCTTACC
AACCTCGGTGCTCCTGCTGCTTTCAAGTACTTCGATACAACCATCGATAGGAAGAGATACACCTCTACCAAAGAAGTGCTCGATGCTACCCTCA
TCCATCAGTCTATCACTGGACTCTACGAGACTAGGATCGATCTCTCACAGCTCGGTGGTGATTCAAGGGCTGATCCTAAGAAGAAGAGGAAGGT
TTGA
```

Supplementary Figure S1B. Sequence information of ^{At}SpCas9, derived from pDe-CAS9 (Fauser et al., 2014). Underlined is the SV40 NLS.

References

Fauser F, Schiml S, Puchta H (2014) Both CRISPR/Cas-based nucleases and nickases can be used efficiently for genome engineering in *Arabidopsis thaliana*. Plant J 79:348–359

ATGGCTCCTAAGAAGAAGCGGAAGGTTGGTATTCACGGGGTGCCTGCGGCTATGGACAAGAAGTACTCGATCGGGCTGGACATCGGAACAAATT
CTGTAGGCTGGGCTGTAATAACCGATGAGTACAAGGTGCCCTCTAAAAAATTTAAGGTCCTTGGCAATACGGATAGACATTCCATAAAGAAGAA
TCTTATCGGTGCGCTGCTCTTTGACAGCGGCGAGACCGCGGAGGCGACCCGGTTGAAACGCACCGCGAGACGCCGTTACACAAGGCGTAAGAAT
AGAATCTGTTATCTCCAGGAGATATTCTCTAATGAAATGGCGAAGGTAGACGATTCCTTCTTTCACCGTCTGGAGGAAAGTTTTCTCGTTGAGG
AAGATAAGAAACATGAAAGACACCCGATCTTCGGAAACATTGTCGACGAGGTCGCTTATCATGAAAAGTACCCTACCATCTACCATCTTAGAAA
GAAACTTGTTGACAGCACGGATAAGGCTGATCTCAGGCTGATATACCTGGCTCTGGCACATATGATTAAGTTCAGAGGGCATTTCCTTATCGAA
GGCGACCTGAATCCAGATAATTCAGATGTAGACAAGCTCTTCATTCAACTTGTGCAGACTTATAATCAGCTCTTCGAAGAAAATCCAATAAACG
CGTCGGGTGTAGACGCAAAGGCCATACTGTCCGCTAGGCTTTCTAAGTCACGTAGACTTGAGAATCTCATTGCCCAACTCCCCGGCGAGAAGAA
GAACGGCTTGTTTGGAAATCTGATAGCGCTGTCCCTGGGTCTTACACCAAATTTCAAGAGTAATTTCGATTTGGCAGAAGATGCTAAGTTGCAG
CTCAGTAAAGACACCTACGATGACGATCTTGATAATTTGTTGGCTCAGATTGGCGATCAGTATGCAGATCTTTTCTTGGCCGCTAAGAATTTGT
CTGATGCAATTCTGCTTAGCGACATTTTGAGGGTTAATACAGAAATCACCAAGGCACCCTTGTCGGCGTCAATGATAAAGAGGTATGATGAGCA
CCACCAAGACCTGACGCTCCTCAAGGCTCTTGTTCGGCAGCAATTGCCGGAGAAGTACAAAGAGATCTTCTTCGACCAGTCTAAGAACGGATAT
GCGGGCTACATAGACGGTGGAGCGAGTCAGGAGGAATTCTACAAGTTCATAAAGCCCATTCTCGAGAAGATGGATGGTACGGAAGAACTGCTTG
TGAAACTTAACAGAGAAGATCTTTTGCGGAAGCAGAGAACTTTCGACAACGGAAGTATACCACACCAGATACATCTCGGAGAGCTTCATGCTAT
TCTCAGAAGACAAGAGGATTTCTACCCTTTCTTGAAGGATAACAGAGAAAAGATAGAGAAGATCCTCACGTTTAGGATCCCTTACTACGTAGGT
CCTCTTGCTCGCGGCAATAGTAGGTTCGCCTGGATGACCCGCAAGTCTGAAGAAACTATCACCCCTTGGAATTTCGAAGAGGTTGTAGACAAAG
GTGCTTCAGCACAGAGTTTCATTGAGAGGATGACCAACTTCGACAAGAACCTCCCCAACGAAAAGGTCCTGCCTAAGCACAGCCTCCTCTACGA
ATACTTTACTGTCTATAATGAGCTTACAAAAGTTAAGTACGTGACAGAGGGAATGCGGAAGCCCGCATTCCTTTCCGGAGAACAAAAGAAGGCG
ATCGTGGATCTTCTCTTCAAGACGAACCGCAAGGTGACGGTTAAACAGTTGAAGGAAGATTACTTCAAGAAGATAGAATGTTTTGATAGCGTGG
AAATCAGCGGCGTCGAAGATAGGTTCAACGCTTCCCTGGGAACGTACCACGATCTCCTCAAGATTATCAAAGATAAGGACTTTCTTGATAACGA
AGAGAATGAGGACATCTTGGAAGACATTGTTCTGACGCTCACCCTGTTCGAAGATCGCGAGATGATTGAGGAACGCTTGAAGACCTACGCACAC
CTGTTCGATGACAAGGTTATGAAGCAACTTAAACGGCGCCGGTATACGGGCTGGGGACGGCTTTCGCGGAAGCTGATAAATGGAATCCGTGACA
AGCAGTCTGGCAAGACAATACTCGACTTCTTGAAGTCGGATGGTTTTGCCAATAGAAATTTTATGCAACTCATTCATGATGACTCGCTTACTTT
TAAGGAGGACATCCAGAAGGCCCAGGTATCAGGACAGGGTGACTCTTTGCACGAACACATCGCGAACCTGGCGGGCTCCCCCGCGATTAAGAAG
GGAATTTTGCAGACTGTCAAGGTGGTCGATGAACTCGTGAAGGTTATGGGACGTCATAAGCCGGAAAATATTGTGATTGAGATGGCTCGCGAGA
ATCAAACAACACAGAAGGGCCAAAAGAACAGTAGAGAACGCATGAAGCGCATCGAAGAGGGCATCAAAGAGCTGGGCAGTCAGATCCTTAAAGA
ACATCCAGTCGAGAATACACAGCTTCAGAACGAAAAGCTGTACCTTTATTACCTTCAAAATGGGCGTGATATGTATGTGGATCAGGAACTCGAT
ATCAATAGGCTGAGTGACTATGATGTCGACCATATCGTCCCGCAAAGTTTCCTCAAGGACGACAGTATAGACAACAAAGTTCTCACACGGTCAG
ATAAGAATCGCGGCAAGAGCGATAATGTACCGTCGGAGGAGGTAGTCAAGAAGATGAAGAATTACTGGCGCCAGTTGCTCAACGCCAAGCTCAT
CACTCAGAGGAAATTTGACAACCTTACGAAAGCCGAGCGGGGCGGACTCTCTGAACTGGACAAGGCCGGTTTCATAAAGCGCCAGCTCGTTGAG
ACACGTCAAATTACTAAGCACGTCGCTCAAATATTGGATTCCCGCATGAATACTAAGTACGATGAGAATGATAAGCTCATACGTGAAGTTAAGG
TCATTACTCTCAAGTCCAAGCTTGTATCGGACTTCCGTAAGGACTTCCAATTCTACAAGGTCCGGGAAATCAATAATTATCACCATGCCCATGA
CGCTTATCTGAACGCGGTCGTGGGCACGGCACTCATTAAGAAATACCCAAAACTTGAGTCAGAATTTGTTTACGGGGACTATAAAGTTTATGAC
GTGCGGAAGATGATAGCGAAGTCGGAACAAGAGATAGGAAAGGCGACTGCAAAGTACTTTTTTTACTCCAACATAATGAATTTCTTTAAGACCG
AAATAACCCTTGCAAACGGTGAAATCAGAAAGCGGCCTCTGATTGAAACAAATGGCGAGACGGGCGAGATCGTCTGGGACAAGGGGAGGGACTT
CGCAACGGTTCGCAAGGTCCTTAGCATGCCGCAAGTAAATATAGTTAAGAAGACGGAAGTTCAGACCGGCGGCTTTAGTAAAGAAAGCATACTT
CCTAAAAGGAATTCCGACAAACTGATAGCGCGCAAGAAGGACTGGGATCCAAAGAAGTATGGAGGATTTGACTCCCCAACCGTTGCTTATAGCG
TGTTGGTAGTAGCCAAGGTGGAAAAGGGTAAGTCTAAGAAATTGAAGTCGGTGAAGGAGTTGTTGGGGATAACTATAATGGAGCGGAGTTCGTT
CGAGAAGAACCCAATTGACTTTCTCGAAGCCAAAGGCTACAAGGAGGTCAAGAAGGACCTGATTATTAAGTTGCCAAAGTACTCGCTCTTCGAA
CTCGAGAACGGGAGAAAGCGTATGCTGGCGTCGGCGGGCGAGCTGCAGAAAGGAAACGAGCTGGCTTTGCCATCGAAATACGTAAATTTCCTGT
ACCTCGCCTCACATTATGAGAAGCTTAAAGGGTCTCCAGAAGACAATGAACAGAAGCAGCTGTTTGTTGAACAGCACAAGCACTACTTGGACGA
GATTATAGAACAAATCTCCGAGTTCTCTAAACGGGTTATCCTTGCAGACGCCAATTTGGATAAGGTCCTCTCGGCTTATAATAAGCATAGAGAT
AAGCCAATCCGGGAGCAGGCTGAAAATATCATACACCTCTTTACGTTGACTAATTTGGGTGCGCCAGCGGCATTCAAGTACTTCGATACAACAA
TCGATCGTAAGCGCTACACAAGCACTAAGGAAGTCCTGGACGCGACGCTGATACACCAGTCCATTACTGGACTGTATGAAACCAGAATAGATCT
TAGCCAGCTCGGCGGTGATTGA

Supplementary Figure S1C. Sequence information of ^{Os}SpCas9, derived from the MMCas9 vector (Mikami et al., 2015). Underlined is the NLS as described before (Cong et al., 2013). The translational enhancer sequence of the 5'-UTR from the *O. sativa* alcohol dehydrogenase 2 gene originally adjacent to the MMCas9 πvector was removed for this study.

References

Cong L, Ran FA, Cox D, Lin S, Barretto R, Habib N, Hsu PD, Wu X, Jiang W, Marraffini LA, et al (2013) Multiplex Genome Engineering Using CRISPR/Cas Systems. Science 339:819–823

Mikami M, Toki S, Endo M (2015) Comparison of CRISPR/Cas9 expression constructs for efficient targeted mutagenesis in rice. Plant Mol Biol 88:561–572

```
   M  D  K  K  Y  S  I  G  L  D  I  G  T  N  S  V  G  W  A  V  I  T  D  E  Y  K  V  P  S  K  K  F  K  V  L  G  N  T  D  R
Cj ATGGACAAGAAGTACAGCATCGGCCTGGATATCGGCACAAATTCTGTTGGCTGGGCCGTGATTACCGACGAGTATAAGGTTCCCAGCAAGAAGTTCAAGGTCCTGGGCAACACAGACAGG  120
At .....T.........TCT....A..C.......A..T..C....G..A....T....C....T....C....G..ATCT............T..C..A.....C..T...  120
Os ..............TCG....G....C....A.........A.......T..A..A....T....C....G...TCT..A..A..T.......T.....T..G..T..A  120

   H  S  I  K  K  N  L  I  G  A  L  L  F  D  S  G  E  T  A  E  A  T  R  L  K  R  T  A  R  R  R  Y  T  R  R  K  N  R  I  C
Cj CACAGCATTAAGAAGAATCTGATTGGCGCCCTGCTGTTCGATTCTGGTGAAACAGCAGAGGCAACAAGGCTGAAGAGGACAGCTAGAAGAAGGTACACCAGGCGTAAGAACAGGATCTGC  240
At ...TCT..C.....A..C..T..C..T..T..C..C................T..T.....T..C..A..C.....A..C..................AA.A..............  240
Os ..TTC...A..........T..C..T..G.....C..T..CAGC..C..G..C..G.....G..CC..T....AC.C..C..G...C.CC.T.....A..........T..A.....T  240

   Y  L  Q  E  I  F  S  N  E  M  A  K  V  D  D  S  F  F  H  R  L  E  E  S  F  L  V  E  E  D  K  K  H  E  R  H  P  I  F  G
Cj TACCTGCAAGAGATCTTCAGCAACGAGATGGCCAAGGTGGACGACAGCTTTTTCCATAGGCTGGAAGAGAGCTTCCTGGTCGAAGAGGATAAGAAGCACGAGAGGCATCCTATCTTCGGC  360
At .....C...........TCT..........T..A.....T..TTCA..C.....C.....C......TCA.....C..G.....A..................C..........A  360
Os ..T..C..G.....A...TCT..T..A.....G.....A.....TTC...C..T..CC.T.....G..A..T..T..C..T..G..A........A..T..A..A..C..G.......A  360

   N  I  V  D  E  V  A  Y  H  E  K  Y  P  T  I  Y  H  L  R  K  K  L  V  D  S  T  D  K  A  D  L  R  L  I  Y  L  A  L  A  H
Cj AACATTGTGGATGAGGTGGCATACCACGAGAAGTACCCTACAATCTACCACCTGAGGAAGAAGCTGGTTGACAGCACAGATAAGGCAGACCTGAGGCTGATCTATCTGGCACTGGCACAC  480
At .....C..T..............................T..........C..A........C.....TTCT..T........T..T..C.....C.....C..C..T..C..T...  480
Os .......C..C.....C..T..T..T..A..........C........T..T..A.....A..T..........G........T..T..C........A..C.....T.......T  480

   M  I  K  F  R  G  H  F  L  I  E  G  D  L  N  P  D  N  S  D  V  D  K  L  F  I  Q  L  V  Q  T  Y  N  Q  L  F  E  E  N  P
Cj ATGATTAAGTTCAGGGGCCACTTTCTGATCGAGGGCGATCTGAATCCCGACAACTCTGATGTGGACAAGCTGTTCATTCAGCTGGTGCAGACATACAACCAGCTGTTCGAAGAGAACCCC  600
At .....C........A..A.....C..C........T.....C..C..T..T.............T...T.......C.....C........C..........T.............T  600
Os .............A..G..T..C..T.....A.....C........A..T..T..A.....A........C........A..T........T..T..T.....C........A..T..A  600

   I  N  A  S  G  V  D  A  K  A  I  L  S  A  R  L  S  K  S  R  R  L  E  N  L  I  A  Q  L  P  G  E  K  K  N  G  L  F  G  N
Cj ATCAACGCATCTGGTGTGGATGCAAAGGCAATTCTGTCTGCCAGGCTGAGCAAGTCAAGAAGGTTGGAGAACCTGATTGCACAGTTGCCTGGCGAGAGAAGAATGGCCTGTTCGGAAAC  720
At ........T..A..........T.....T..C..C.....T.....CTCT...........C.T.......C.....T...C.C.....T...........C..A..T.........  720
Os ..A.....G..G.....A..C........C..A.....C..T.....TTCT......C.T..AC.T.....T..C.....C..AC.C..C..............C...T....T.....T  720

   L  I  A  L  S  L  G  L  T  P  N  F  K  S  N  F  D  L  A  E  D  A  K  L  Q  L  S  K  D  T  Y  D  D  D  L  D  N  L  L  A
Cj CTCATTGCACTGTCTTTGGGCCTGACACCTAACTTCAAGAGCAACTTCGATCTGGCCGAGGATGCTAAGTTGCAGCTGTCTAAGGATACCTACGATGACGACCTGGATAACCTGCTGGCA  840
At T.G..C..T..C...C.C..A..C..C...........TCT..........C..T........A...C.C.....C..A................T..T..C........C..C..T  840
Os ..G..A..G.....CC....T..T.....A..T........T..T......T....A..A.................CAG...A..C..............T..T.....TT..T....T  840

   Q  I  G  D  Q  Y  A  D  L  F  L  A  A  K  N  L  S  D  A  I  L  L  S  D  I  L  R  V  N  T  E  I  T  K  A  P  L  S  A  S
Cj CAGATTGGAGATCAGTACGCAGATCTGTTCCTGGCAGCAAAGAACCTGTCTGATGCCATCCTGCTGAGCGATATTCTGAGGGTGAACACCGAGATTACAAAGGCCCCTCTGAGCGCCTCT  960
At .....C..............T...T.......C..T..T.......C........T.....C..C..T.....C..C..A..............C..C.....T..A..CTCA..T...  960
Os ........C........T........T...T....C..T.....TT..........A..T.....T.....C...T.......T..T..A..A..C..C.....A..CT..TCG..G..A  960

   M  I  K  R  Y  D  E  H  H  Q  D  L  T  L  L  K  A  L  V  R  Q  Q  L  P  E  K  Y  K  E  I  F  F  D  Q  S  K  N  G  Y  A
Cj ATGATCAAGAGATACGATGAGCACCACCAGGATCTGACATTGCTGAAGGCATTGGTTAGGCAGCAGCTGCCAGAGAAGTACAAAGAGATTTTCTTCGATCAGAGCAAGAACGGCTACGCC  1080
At .................................C...C..T..C.....TC..T.....A........C...............................TCT........A.....T  1080
Os .....A.....G..T.............A..C.....GC..C..C.....TC..T...C.......AT....G................C........C...TCT........A..T..G  1080

   G  Y  I  D  G  G  A  S  Q  E  E  F  Y  K  F  I  K  P  I  L  E  K  M  D  G  T  E  E  L  L  V  K  L  N  R  E  D  L  L  R
Cj GGCTATATTGATGGTGGTGCATCTCAAGAAGAGTTCTACAAGTTCATCAAGCCCATCCTCGAGAAGATGGATGGCACAGAGGAATTGCTGGTGAAGCTGAACAGAGAGGACTTGTTGAGG  1200
At ..T..C..C.......................................T...................A..C......C..C..C.......C..T........TC.TC..C..A  1200
Os .....C..A..C.....A..GAG...G..G..A.............A........T................T..G..A...C....T.....A..T........A..TC..T...C..  1200

   K  Q  R  T  F  D  N  G  S  I  P  H  Q  I  H  L  G  E  L  H  A  I  L  R  R  Q  E  D  F  Y  P  F  L  K  D  N  R  E  K  I
Cj AAGCAGAGGACCTTCGATAACGGCAGCATTCCTCATCAGATTCATCTGGGAGAGCTGCACGCAATCTTGAGAAGGCAAGAGGACTTCTACCCATTCCTGAAGGACAACCGTGAGAAGATA  1320
At ......................ATCT..C..........C..C..C......T.......T...C.T.............T.............C.....T...A..G..A.....T  1320
Os ........A..T.....C.....A..T..A..A..C.....A.....C........T..T..T..TC.C.....A........T........T...T.......T...A..A..A......  1320

   E  K  I  L  T  F  R  I  P  Y  Y  V  G  P  L  A  R  G  N  S  R  F  A  W  M  T  R  K  S  E  E  T  I  T  P  W  N  F  E  E
Cj GAGAAGATCCTGACCTTCAGGATCCCCTATTATGTGGGACCACTGGCAAGGGGCAATTCTAGGTTTGCTTGGATGACCCGTAAGAGCGAAGAGACAATTACCCCTTGGAACTTCGAAGAG  1440
At ........T..C........A.....T..C..C.......T..C..T..A..A..C..A..A..C............A.A...TCT..G..A..C..C....................  1440
Os ...........C..G..T........T..C..C..A..T..T..T..TC.C......AG......C..C...........C...TCT.....A..T..C............T.........  1440

   V  V  D  K  G  A  S  A  Q  S  F  I  E  R  M  T  N  F  D  K  N  L  P  N  E  K  V  L  P  K  H  S  L  L  Y  E  Y  F  T  V
Cj GTGGTGGATAAGGGCGCATCTGCCCAGTCTTTTATTGAGAGGATGACCAACTTCGACAAGAACCTGCCTAACGAGAAGGTGCTGCCAAAGCACTCTCTGCTGTACGAGTACTTCACCGTG  1560
At .............T..TAG...T.......C..C...................T........T..A.............C..T.........T....C.................  1560
Os ..T..A..C..A..T..T..A..A...AG...C...............................C..C.....A.....C.....T......AGC..C..C.....A.....T..T..C  1560

   Y  N  E  L  T  K  V  K  Y  V  T  E  G  M  R  K  P  A  F  L  S  G  E  Q  K  K  A  I  V  D  L  L  F  K  T  N  R  K  V  T
Cj TACAACGAGCTGACCAAAGTGAAGTATGTGACCGAGGGCATGAGAAAGCCTGCATTTTTGTCTGGGGAGCAGAAGAAGGCCATCGTCGATTTGCTGTTCAAGACCAACAGAAAGGTGACC  1680
At .........T.......G..T.....C..........A.....G........T........A..T....A........T.....T...C.CT........................  1680
Os ..T..T.....T..A.....T.....C.....A.....A...C.G.....C.....CC.T..C..A..A..A........G.....G...C..T..C........G...C..C.......G  1680

   V  K  Q  L  K  E  D  Y  F  K  K  I  E  C  F  D  S  V  E  I  S  G  V  E  D  R  F  N  A  S  L  G  T  Y  H  D  L  L  K  I
Cj GTGAAGCAGCTGAAAGAGGACTACTTCAAGAAAATCGAGTGCTTCGACAGCGTCGAGATCTCTGGTGTTGAGGATAGGTTCAATGCCAGCCTGGGCACATATCACGACCTGCTCAAGATC  1800
At ...........C........T........................TTCA..T.....T.......................C..ATCT..C..A..C..C.....T..C.........  1800
Os ..T..A...T....G..A..T...........G..A..A..T..T..T.....G..A...AGC..C..C..A...........C..TTC......A..G..C.....T..C........T  1800

   I  K  D  K  D  F  L  D  N  E  E  N  E  D  I  L  E  D  I  V  L  T  L  T  L  F  E  D  R  E  M  I  E  E  R  L  K  T  Y  A
Cj ATCAAGGACAAGGACTTTCTGGACAACGAGGAAAACGAGGACATCTTGGAGGATATCGTGCTGACACTGACCCTCTTCGAGGATAGAGAGATGATCGAGGAAAGGCTCAAGACATACGCA  1920
At ..T.....T.....T..CT....T................T................T..T..C..C........T..A..............T..A..............C.....T  1920
Os .....A..T..........T..T.....A..G..T.............A..C..T..T.....G..C.....G.....A...C.C........T......C.CT.G.....C......  1920

   H  L  F  D  D  K  V  M  K  Q  L  K  R  R  R  Y  T  G  W  G  R  L  S  R  K  L  I  N  G  I  R  D  K  Q  S  G  K  T  I  L
Cj CACCTGTTCGACGACAAGGTGATGAAGCAGTTGAAGCGTAGAAGGTATACAGGCTGGGGCAGACTGTCACGTAAGCTGATTAACGGCATCAGGGATAAGCAGAGCGGCAAGACCATTCTC  2040
At ..T..C.....T..T....................A.A.....A..C..T..T.....A..G..C...A.A.....C........A...............TCT..A.....A..C..T  2040
Os ..........T........T........AC.T..A..GC.CC.......G........AC.G..T..G..G........A..T..A...C.T..C......TCT........A..A...  2040

   D  F  L  K  S  D  G  F  A  N  R  N  F  M  Q  L  I  H  D  D  S  L  T  F  K  E  D  I  Q  K  A  Q  V  S  G  Q  G  D  S  L
Cj GACTTTCTGAAGTCTGATGGCTTCGCCAACCGTAACTTCATGCAGCTGATCCACGATGACAGCCTCACCTTCAAAGAGGATATCCAGAAGGCACAGGTTAGCGGACAAGGCGATTCTCTG  2160
At ..T..C..C..........A.....T..A.A.............C..........TTCT.......T....................T......TCA.....G..T...AG...C  2160
Os .....CT.......G.....T..T.....TA.A..T..T.....A..C..T..T......TCG..T..T..T..G.....C...........C.....ATCA.....G..T..C...T..  2160

   H  E  H  I  A  N  L  A  G  S  P  A  I  K  K  G  I  L  Q  T  V  K  V  V  D  E  L  V  K  V  M  G  R  H  K  P  E  N  I  V
Cj CATGAGCACATTGCAAATCTGGCAGGCAGCCCTGCTATTAAGAAGGGCATTCTGCAGACAGTGAAGGTCGTGGATGAGTTGGTTAAGGTGATGGGCAGACACAAGCCTGAGAACATCGTG  2280
At ........T..C..T..C..C..T..ATCT.....A..C........A..C..C.....T........T.............G..........A..G..T.................  2280
Os ..C..A.....C..G..C.....G...TC...C..G...........A...T........T..C.....G..C.....AC.C..G.....T.....AC.T..T.....G..A..T..T...  2280
```

```
    I  E  M  A  R  E  N  Q  T  T  Q  K  G  Q  K  N  S  R  E  R  M  K  R  I  E  E  G  I  K  E  L  G  S  Q  I  L  K  E  H  P
Cj ATTGAGATGGCACGTGAGAACCAGACAACTCAGAAGGGCCAGAAGAACTCCAGGGAACGTATGAAGAGGATTGAAGAGGGCATCAAAGAGCTGGGCTCCCAGATTTTGAAAGAGCACCCT  2400
At ..C..A.....TA.A..........C..........A..........T.....A.G..........C..G..A..T..........T..A..T.....CC.C...........  2400
Os ..........T..C.....T..A.....A..........A......AGT..A.....C......C.C..C.......................AGT.....CC.T.....A..T..A  2400

    V  E  N  T  Q  L  Q  N  E  K  L  Y  L  Y  Y  L  Q  N  G  R  D  M  Y  V  D  Q  E  L  D  I  N  R  L  S  D  Y  D  V  D  H
Cj GTTGAGAACACCCAGCTGCAGAACGAGAAGCTGTATCTGTACTACTTGCAGAACGGCAGGGACATGTATGTGGATCAAGAGCTGGATATCAACAGGCTGTCCGATTACGACGTGGACCAT  2520
At ..........T.....C.....T........C..C..C......C.C........A.....T..................T...............C..T........T..T..T...  2520
Os ..C.....T..A.....T........A........C..T..T...C.T..A..T..GC.T..T.............G..A..C........T......AGT..C..T..T..C......  2520

    I  V  P  Q  S  F  L  K  D  D  S  I  D  N  K  V  L  T  R  S  D  K  N  R  G  K  S  D  N  V  P  S  E  E  V  V  K  K  M  K
Cj ATTGTGCCTCAGAGCTTCTTGAAGGACGACTCCATCGATAACAAGGTGCTGACCAGGTCCGATAAGAACAGAGGCAAGAGCGATAATGTGCCCTCCGAAGAGGTTGTGAAGAAGATGAAG  2640
At ..C.....A...TCA..........T..T..T................C........T..........G..T.....T.....C.....AAGT................A......  2640
Os ..C..C..G..A..T...C.C.........AGT..A..C.....A..T..C..AC....A........TC.C.................A..G..G..G.....A..C...........  2640

    N  Y  W  R  Q  L  L  N  A  K  L  I  T  Q  R  K  F  D  N  L  T  K  A  E  R  G  G  L  S  E  L  D  K  A  G  F  I  K  R  Q
Cj AACTACTGGCGTCAGCTCCTGAACGCCAAGTTGATTACTCAGAGGAAGTTCGACAACCTGACCAAGGCAGAAAGAGGCGGATTGAGCGAATTGGATAAGGCCGGCTTTATTAAGAGGCAG  2760
At .....T...A.G........C.....T...C.C..C........A........T...T....T.....T..G..G..A...C.CTCT.............A..A..C..C.........  2760
Os ..T........C...T.G..C.........C.C..C...........A..T........T..G..A..C..GC.G......C.CTCT...C....C........T..C..A...C.C...  2760

    L  V  E  T  R  Q  I  T  K  H  V  A  Q  I  L  D  S  R  M  N  T  K  Y  D  E  N  D  K  L  I  R  E  V  K  V  I  T  L  K  S
Cj CTGGTGGAAACAAGGCAGATCACAAAACACGTGGCCCAGATTCTGGACTCCAGGATGAACACAAAGTACGACGAGAATGACAAGCTGATCCGTGAGGTGAAGGTCATCACCCTGAAGTCT  2880
At ..T........C..........T..G.....T..A.....C..C..T..T...........C........T.....C..T...T.....A.G..A........T........C.....A  2880
Os ..C..T..G...C.T..A..T..T..G.....C..T..A..AT....T...C.C.....T..T........T........T.....C..A.....A..T........T..T..C.....C  2880

    K  L  V  S  D  F  R  K  D  F  Q  F  Y  K  V  R  E  I  N  N  Y  H  H  A  H  D  A  Y  L  N  A  V  V  G  T  A  L  I  K  K
Cj AAGCTGGTGAGCGACTTCCGTAAGGACTTCCAGTTCTATAAGGTGCGAGAGATCAACAACTACCACCACGCACATGATGCCTACCTGAATGCAGTTGTGGGCACAGCACTGATCAAGAAG  3000
At .....C...TCT..T...A.A.....T.....A.....C.....A.G..A..................T..C.....T.....T..C..T.....T..A..C..T..C.........  3000
Os .....T..ATCG...................A.....C.....C..G..A.....T..T..T.....T..C.....C..T..T.....C..G..C........G.....C..T.....A  3000

    Y  P  K  L  E  S  E  F  V  Y  G  D  Y  K  V  Y  D  V  R  K  M  I  A  K  S  E  Q  E  I  G  K  A  T  A  K  Y  F  F  Y  S
Cj TATCCCAAGCTCGAGAGCGAGTTCGTGTACGGTGATTACAAGGTGTACGACGTGAGGAAGATGATCGCCAAGAGCGAGCAAGAGATTGGCAAGGCAACAGCCAAGTACTTCTTCTACAGC  3120
At .....T.........TCA..............................T................T...TCT..........C..A.....T..C..T.....T.........TCT  3120
Os ..C..A..A..T...TCA..A..T..T.....G..C..T..A..T..T......C..........A..G...TCG..A........A..A.....G..T..A........T..T...TC.  3120

    N  I  M  N  F  F  K  T  E  I  T  L  A  N  G  E  I  R  K  R  P  L  I  E  T  N  G  E  T  G  E  I  V  W  D  K  G  R  D  F
Cj AACATCATGAATTTCTTCAAGACCGAGATCACCCTGGCCAACGGCGAAATTAGGAAGAGGCCTTTGATTGAGACAAACGGCGAGACAGGTGAGATCGTGTGGGATAAGGGTAGAGATTTC  3240
At ............................T.....C..T.....T..G..C..A........AC.C..C...........T..A..........................A..G......  3240
Os .....A...........T........A..A.....T..A.....T.....C..A...C.....C.......A.....T........G..C........C.....C.....G..G..C...  3240

    A  T  V  R  K  V  L  S  M  P  Q  V  N  I  V  K  K  T  E  V  Q  T  G  G  F  S  K  E  S  I  L  P  K  R  N  S  D  K  L  I
Cj GCCACAGTGCGTAAGGTGCTGTCTATGCCTCAGGTGAACATTGTGAAAAAGACCGAGGTTCAGACCGGCGGCTTCAGCAAAGAGTCTATTCTGCCTAAGAGGAACAGCGATAAGCTGATC  3360
At ..T..C..TA.A........C........A...........C..T..G..A........G........T..A...TCT...........C..C............TCT........C..T  3360
Os ..A..G..T..C.....C..TAGC.....G..A..A..T..A..T..G.....G..A................T..T.....AAGC..A..T.....A.....TTC...C..A.....A  3360

    A  R  K  K  D  W  D  P  K  K  Y  G  G  F  D  S  P  T  V  A  Y  S  V  L  V  V  A  K  V  E  K  G  K  S  K  K  L  K  S  V
Cj GCCAGAAAGAAGGATTGGGACCCTAAGAAGTATGGCGGCTTCGATTCTCCTACAGTGGCATATTCTGTGCTGGTGGTGGCAAAGGTTGAGAAGGGGAAGTCAAAGAAGCTGAAGTCCGTC  3480
At ..T..G......................A..C..T..T.............C.....T..C.....T..C..T.....T.............A...AGT........C.....T..T  3480
Os ..GC.C........C.....T..A...........A..A..T..C..C..A..C..T..T...AGC...T....A..A..C.....G..A.....T.....T.....AT.......G..G  3480

    K  E  L  L  G  I  T  I  M  E  R  S  S  F  E  K  N  P  I  D  F  L  E  A  K  G  Y  K  E  V  K  K  D  L  I  I  K  L  P  K
Cj AAAGAGTTGCTGGGCATCACAATCATGGAAAGGTCCAGCTTCGAGAAGAATCCTATCGACTTCTTGGAGGCCAAGGGCTACAAAGAGGTGAAGAAGGACCTCATCATCAAGCTGCCCAAG  3600
At ..G..AC.T..C..A.....T.............ATCT..........C..A.....T...C.C.....T.....A...........T........T.............C..A...  3600
Os ..G......T....G..A..T..A.....GC..AGTTCG...........C..A..T.....TC.C..A.....A........G.....C...........G..T..T...T....A...  3600

    Y  S  L  F  E  L  E  N  G  R  K  R  M  L  A  S  A  G  E  L  Q  K  G  N  E  L  A  L  P  S  K  Y  V  N  F  L  Y  L  A  S
Cj TACTCACTGTTCGAGTTGGAGAATGGCCGTAAGAGGATGTTGGCATCTGCTGGCGAATTGCAGAAGGGAAACGAATTGGCACTGCCCTCCAAGTACGTGAACTTCCTGTATCTGGCAAGC  3720
At ........C.....AC.C.....C..TA.A.........C.C..T........T..GC.T..A...........GC.T..T..C..A..T........T.....T..T..C..C..TTCT  3720
Os .....G..C.....AC.C.....C..GA.A...C.T...C.....G..G..G.....GC.......A.........GC....TT....A..G..A.....A..T........C..C..CTCA  3720

    H  Y  E  K  L  K  G  S  P  E  D  N  E  Q  K  Q  L  F  V  E  Q  H  K  H  Y  L  D  E  I  I  E  Q  I  S  E  F  S  K  R  V
Cj CACTACGAGAAGTTGAAGGGCTCTCCTGAGGATAACGAGCAGAAGCAGCTTTTCGTCGAGCAGCATAAGCACTACCTGGACGAGATTATCGAGCAGATCAGCGAGTTCAGCAAGAGGGTG  3840
At ...................A.....A..A.................A........T.......C.........T....T.....C...........TCT......TCT..A......  3840
Os ..T..T......C.T..A..G.....A..A..C..T..A...........G..T..T..A.....C.........T.............A..A..A...TC.......TCT..AC....T  3840

    I  L  A  D  A  N  L  D  K  V  L  S  A  Y  N  K  H  R  D  K  P  I  R  E  Q  A  E  N  I  I  H  L  F  T  L  T  N  L  G  A
Cj ATACTGGCAGATGCAAACCTGGACAAGGTGTTGAGCGCATACAACAAGCACAGGGACAAGCCTATTAGAGAGCAGGCCGAGAACATCATCCACCTGTTCACACTGACAAATCTGGGTGCT  3960
At ..C..C..T..........C..T.........TCT..T..............A..T........C..G..A.....A..............T..C.....C..T..C..C..C......  3960
Os ..C..T.....C..C..TT....T.....CC.CTCG..T..T..T.....T..A..T.....A..CC.G........T..A..T.....A.....C..T..GT....T...T.......G  3960

    P  A  A  F  K  Y  F  D  T  T  I  D  R  K  R  Y  T  S  T  K  E  V  L  D  A  T  L  I  H  Q  S  I  T  G  L  Y  E  T  R  I
Cj CCTGCAGCCTTCAAGTATTTCGATACCACCATCGACCGAAAGAGGTACACCTCCACAAAAGAGGTGTTGGACGCCACACTGATCCACCAGTCTATTACAGGACTGTACGAGACAAGGATC  4080
At .....T..T........C........A........TA.G.....A........T..C.....A...C.C..T..T..C..C.....T........C..T.....C........T......  4080
Os ..A..G..A........C........A..A.....T..T...C.C.....AAG...T..G..A..CC.......G..G.....A........C.....T........T..A..C..A..A  4080

    D  L  S  Q  L  G  G  D  *
Cj GACCTGTCTCAACTTGGAGGTGATTAG                                                                                               4107
At ..T..C..A..G..C..T.......GA                                                                                               4107
Os ..T..TAGC..G..C..C.......GA                                                                                               4107
```

Supplementary Figure S2. Amino acid sequence of SpCas9 and sequence alignment of the Cas9 genes from ^{Cj}SpCas9 (Cj), ^{At}SpCas9 (At), and ^{Os}SpCas9 (Os). Bold letters indicate amino acid sequences. Dots indicate identical bases.

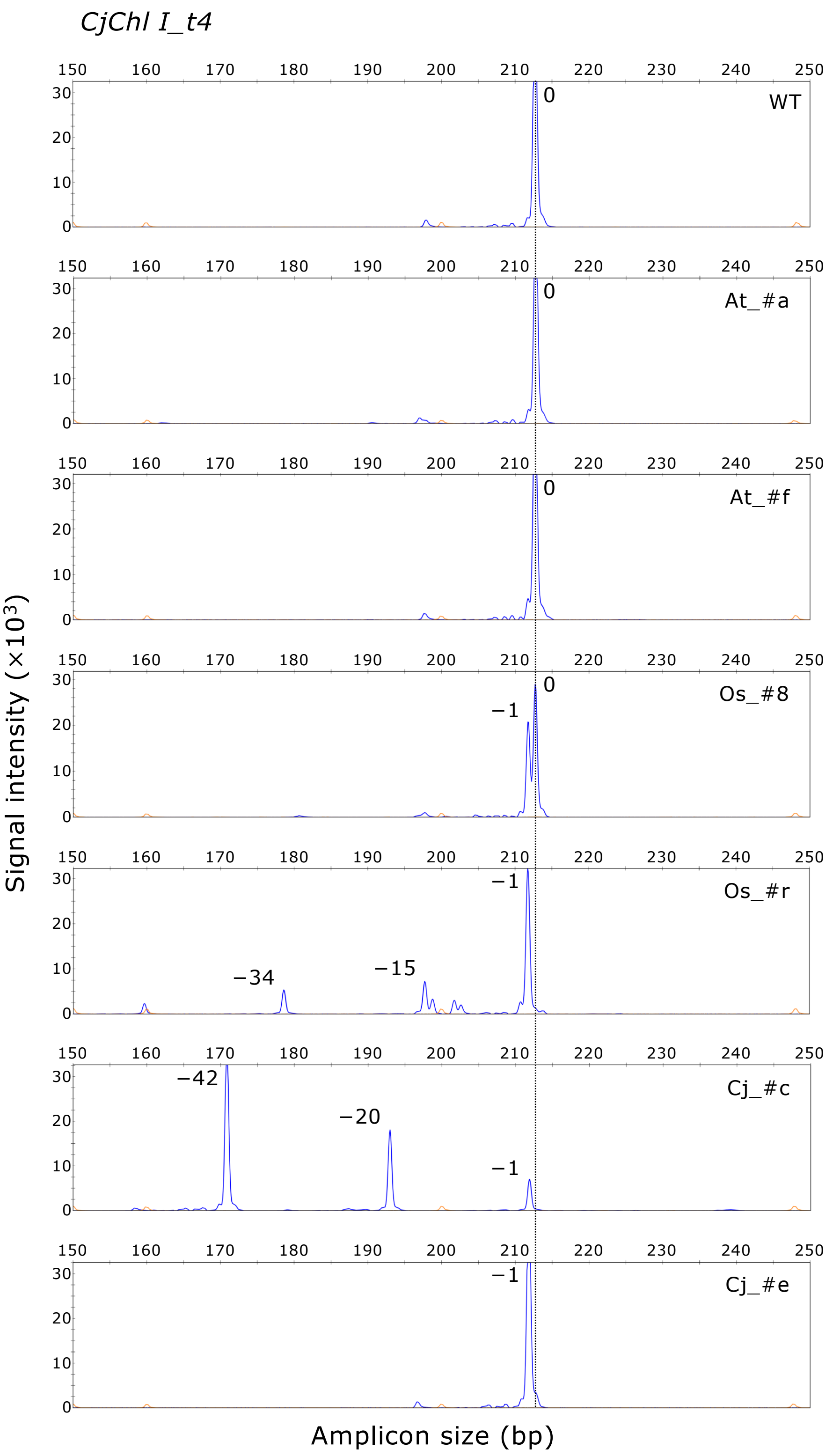

Supplementary Figure S3A. Indel patterns of ET cell lines induced by gene targeting around target 4 in *CjChl I*. Shown from top to bottom are the amplified unedited-WT amplicon peak, pCRG-^{At}SpCas9_CjChl I_t4-introduced lines #a and #f (At_#a and At_#f), pCRG-^{Os}SpCas9_CjChl I_t4-introduced lines #8 and #r (Os_#8 and Os_#r), and pCRG-^{Cj}SpCas9_CjChl I_t4-introduced lines #c and #e (Cj_#c and Cj_#e), respectively. The numbers near the peaks indicate the indel size.

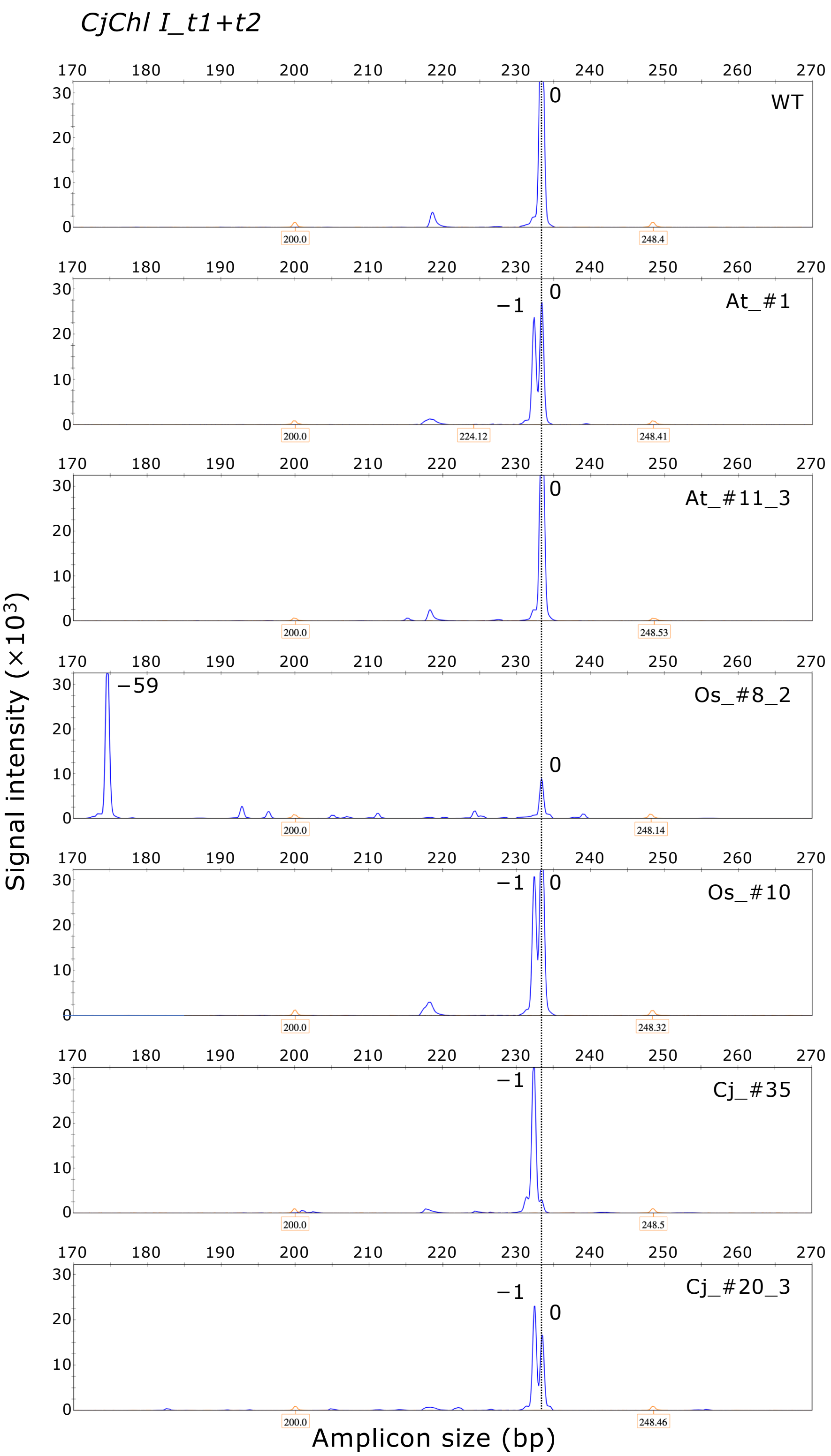

Supplementary Figure S3B. Indel patterns of ET cell lines induced by gene targeting around target 1 and 2 in *CjChl I*. Shown from top to bottom are the amplified unedited-WT amplicon peak, pCRG-[At]SpCas9_CjChl I_t1+t2-introduced lines #1 and #11_3 (At_#1 and At_#11_3), pCRG-[Os]SpCas9_CjChl I_t1+t2-introduced lines #8_2 and #10 (Os_#8_2 and Os_#10), and pCRG-[Cj]SpCas9_CjChl I_t1+t2-introduced lines #35 and #20_3 (Cj_#35 and Cj_#20_3), respectively. The numbers near the peaks indicate the indel size.

Supplementary Table S2. List of oligonucleotides used in this study

Name	Sequence (5'-3')	Application
p#474_f	GCTGAAGCACTGCACGCCGTGTTTTAGAGCTAGAA	gRNA cassette for GFP
p#475_r	ACGGCGTGCAGTGCTTCAGCCACAACAGCAGGCGT	
p#440_f	TGAGTAGTTGACAGCAGCCAGTTTTAGAGCTAGAA	gRNA cassette for *CjChl I*_t4 region
p#441_r	TGGCTGCTGTCAACTACTCAcACAACAGCAGGCGT	
p#353_f	GATTGACCCAAAAATTGGAGGTTTTAGAGCTAGAA	gRNA cassette for *CjChl I*_t1 region
p#354_r	CTCCAATTTTTGGGTCAATCcACAACAGCAGGCGT	
p#355_f	GTTATGATAATGGGTGACCGGTTTTAGAGCTAGAA	gRNA cassette for *CjChl I*_t2 region
p#356_r	CGGTCACCCATTATCATAACcACAACAGCAGGCGT	
p#329_T3_r	AACCCTCACTAAAGGGAAGTTGGGTAACGCCAGGGTTT	gRNA cassette
p#347_T3_f	CCCTTTAGTGAGGGTTAATTTAATTAAGGTACCGACTCG	
p#343_f	TTTAAGCTTGGCGCGCCTTAATTAAGGTACCGAGCTC	
p#333_r	TACGAATTGGGCGCGGTTGGGTAACGCCAGGGTTT	
T7-Fam_f	ATGCTAGTTATTGCTCAGCGG	6-FAM labbeled universal primer
p#568_f	ATGCTAGTTATTGCTCAGCGGAGAGAGCAAGTCATGGCAATGGCAATC	Indel detection for *CjChl I*_t4 region
p#465_r	CTCACGACAAGGCAAGTGC	
p#569_f	ATGCTAGTTATTGCTCAGCGGCTACGAAGGCTGTATCCAAGGACAATC	Indel detection for *CjChl I*_t1 and t2 regions

Supplementary Table S3A Phenotypes of the seedlings genome-edited targeting t4 in *CjChl I* with various SpCas9 genes.

Target	Cas9 type	Line	Mutation pattern					
			ET	Regenerated plants (phenotype)				
				#1	#2	#3	#4	#5
t4	–	WT	0	0 (G)	0 (G)			
	At	a	0	-1 (W)	0, -1 (G)			
		c	0	-1, -4, -15 (W)	0, -1, -3 (G)			
		j	0	0, -1, -3 (W)	0, -3, -15 (G)	0, +2 (G)	-1, +2 (W)	0, -1, -105 (G)
		e	0	0, -1, -15 (G)	0, -1, -18 (G)			
		f	0	0, -1 (G)	0, -2 (G)			
		h	0	0, -15, -23 (G)	0, -3, -15 (G)			
		k	0	0, -3, -14 (G)	0, -3, -14 (G)			
		d	0	0, -3, -14 (G)	ND (G)			
	Os	g	0, -1	-1 (W)	0 (G)			
		p	0, -1	-1, -4, -15 (W)	0, -3 (G)			
		c	0	0, -3, -15 (G)	0, -3, -15 (G)			
		m	0	0, -24, -38 (G)	0, -3, -15 (G)			
		n	0, -3, -14	0, -15 (G)	0, -3, -15 (G)			
	Cj	a	0, -1	-1, -15 (G)	-1, -4, -15 (W)			
		b	-1, +1	-1, -14, +1 (W)	-1, -14, +1 (W)			
		d	0, -2	-2, -5, -17 (W)	-2, -5, -17 (W)			
		e	-1	-1, -4, -16 (W)	-1, -4, -16 (W)			
		h	0, -1, +1	-1, -8 (W)	0, -1, -2, -45 (G)			
		j	0, -1, -15	-15 (G)	-1, -4, -15 (W)			
		c	-1, -20, -42	-42 (G)	-42 (G)			
		f	0, -1	0, -15, +1 (G)	-3 (G)			

G; greenish, W; albino

Supplementary Table S3B Phenotypes of the seedlings genome-edited targeting t1 + t2 in *CjChl I* with various SpCas9 genes.

Target	Cas9 type	Line	Mutation pattern					
			ET	Regenerated plants(phenotype)				
				#1	#2	#3	#4	#5
t1+t2	–	WT	0	0 (G)	0 (G)			
	At	b	0, -1	0, -15 (G)	0, -7 (G)			
		c	0	0, -1 (G)	0, -1 (G)			
		f	0, -1	0, -1 (G)	0, -1 (G)			
		g	0, -1	0 (G)	0, -1 (G)			
		1	0, -1	0, -1 (G)	0, -1 (G)			
		6	0	0 (G)	0, -6, -15 (G)			
	Os	e	0	0 (G)	0 (G)			
		g	0, -9	0 (G)	0 (G)			
		m	0, -1, -16	-1, -2 (W)	0, -27 (G)			
		1	0, -37	0 (G)	0 (G)			
		4	0	0 (G)	0 (G)			
		6	0	+1, -18 (W)	-1, -18 (W)	0, -18 (G)		
		7	0, -24, -37, -59, +18	0 (G)	0 (G)	0 (G)	0, -15 (G)	
		8	0, -1, -23, -37, -41, -59	-9, -59, -71 (W)	-59 (W)	-59, -37 (W)	0, -59 (G)	-27, -59 (W)
		12	0, -22	0 (G)	0 (G)			
	Cj	p	0, -8, -25	-34, -39, -47 (W)	-1. -2 (W)			
		1	0, -25, -26	-1, -2, -8 (W)	0, -1 (G)			
		9	0, -5, -7	0, -27 (G)	0, -1 (G)			
		10	-2, -7, -22, -24	-2, -3, -18 (W)	-2, -3, -18 (W)			
		15	0, -1, -8	0,-1 (G)	0,-1 (G)			
		18	0, -5, -30	0, -2 (G)	-1, -2, -9 (G)			
		20	0, -1	0, -1, -2, -3 (G)	0, -1, -2, -23 (G)			
		23	0, -1	0, -41, -46 (G)	-1, -2 (W)			
		25	0, -1, -24, -41	0, -17, -18 (G)	-1, -36, -37 (W)			
		28	0, -41	0, -16 (G)	0, -15 (G)			
		30	0, -20	0, -1, -2 (G)	0, -1 (G)			
		35	-1	-2, -8, -18 (W)	-1, -2. -3 (W)			

G; greenish, W; albino
